# Supplementary material for: The spatio-temporal evolution of multiple myeloma from baseline to relapse-refractory states
Source: Nat Commun. 2022 Aug 3;13:4517. doi: 10.1038/s41467-022-32145-y (PMC9349320; doi:10.1038/s41467-022-32145-y)

SUPPLEMENTARY INFORMATION

The Spatio-Temporal Evolution of Multiple Myeloma from Baseline to Relapse-Refractory States

Rasche *et al.*

Supplementary Table 1: Evolutionary pattern

ID	Excluded	Single sub-clone expansion	Coexisting sub-clones	Spatial dominance	PET FL	Response	SG	GEP70 baseline
1	-	x	-	-	2	CR	PR	low risk
2	-	-	x	-	140	PR	HY	low risk
3	only one FU sample	NA	NA	NA	masked	CR	PR	high risk
4	only one FU sample	NA	NA	NA	30	CR	t(4;14)	high risk
5	only one FU sample	NA	NA	NA	0	CR	CD-2	low risk
6	-	-	x	-	11	VGPR	HY	low risk
7	-	-	x	-	0	CR	MF	high risk
8	-	-	x	-	35	PR	HY	low risk
9	-	-	x	-	4	VGPR	HY	low risk
10	-	-	-	x	50	CR	MS	high risk
11	-	-	-	x	11	CR	t(6;14)	low risk
12	-	-	x	-	1	VGPR	MS	NA
13	-	-	-	x	21	CR	HY	low risk
14	-	-	x	-	3	PR	HY	low risk
15	-	-	x	-	4	CR	PR	low risk
16	-	-	x	-	2	CR	HY	low risk
17	-	x	-	-	1	CR	CD-1	low risk
18	-	x	-	-	1	CR	LB	low risk
19	-	x	-	-	1	CR	CD-2	low risk
20	-	x	-	-	1	CR	PR	high risk
21	-	x	-	-	0	CR	MS	low risk
22	-	x	-	-	0	CR	HY	low risk
23	-	-	-	x	25	CR	MS	low risk
24	-	-	x	-	20	CR	HY	low risk

Supplementary Table 2: Contribution of SBS-MM1 signature to sub-clonal mutations

ID	Clone ID	# Mutations	SBS-MM1 contribution (mmsig)*	*P* (mSigAct)**	SBS-MM1***
1	1.1	25	0.42 (0-0.62)	1.47E-03	yes
2	1.1.1.1	44	0.43 (0-0.62)	4.33E-05	yes
3	1.1	62	0.56 (0.42-0.68)	1.63E-08	yes
4	1.2	54	0.44 (0.34-0.6)	1.40E-05	yes
5	1.3	34	0.37 (0.09-0.57)	2.79E-04	yes
6	1.1.2	86	0.33 (0.14-0.46)	1.27E-05	yes
7	1.2.1 & 1.2.2 combined	58	0 (0-0.08)	0.22	no
7	1.2.2.1	405	0 (0-0)	3.79E-05	no
9	1.1.2	87	0.48 (0.32-0.62)	1.57E-09	yes
10	1.2.1	126	0.35 (0.26-0.47)	1.72E-06	yes
11	1.2.1.1	83	0.3 (0.09-0.37)	4.84E-06	yes
11	1.2.1.2.2	64	0.52 (0.27-0.65)	7.75E-08	yes
12	1.1.1	53	0.58 (0.43-0.78)	2.13E-09	yes
12	1.1.2	60	0.32 (0.05-0.53)	6.04E-04	yes
12	1.1.3	48	0.32 (0-0.45)	6.46E-04	yes
13	1.2.1.2.1	25	0.41 (0.08-0.61)	8.12E-03	yes
13	1.2.3	70	0.12 (0-0.37)	0.12	no
14	1.1.1.1.1	35	0 (0-0)	1.00	no
15	1.1.1.1	111	0.43 (0.28-0.55)	2.68E-08	yes
15	1.2.1	40	0 (0-0.31)	0.41	no
16	1.2.1	98	0.5 (0.38-0.62)	1.76E-13	yes
16	1.2.2 & 1.2.2.1 combined	52	0.46 (0.3-0.55)	1.79E-05	yes
17	1.2	91	0.21 (0-0.32)	2.50E-02	yes
18	1.2	81	0.23 (0.07-0.33)	1.69E-03	yes
19	1.2.1	48	0 (0-0.28)	0.45	no
19	1.2.1.1	26	0 (0-0.2)	1.00	no
20	1.1.2.2.1	66	0.38 (0.26-0.48)	3.90E-06	yes
21	1.1.1	47	0.19 (0.04-0.44)	3.58E-02	yes
21	1.1.1.1.1	32	0 (0-0)	1.00	no
22	1.1.1.1	80	0.3 (0.12-0.4)	2.83E-05	yes
23	1.2.2	147	0.23 (0.19-0.35)	9.54E-06	yes
23	1.2.3.1	55	0.1 (0-0.28)	0.19	no
23	1.2.3.2	111	0.14 (0.04-0.31)	1.85E-02	yes
23	1.2.3.2.1	35	0.41 (0-0.55)	3.30E-04	yes
24	1.1.1	63	0.28 (0.17-0.5)	3.83E-04	yes
24	2.1	33	0 (0-0.13)	0.81	no
24	2.1.1	46	0.49 (0.3-0.6)	1.08E-08	yes
24	2.1.1.2	34	0 (0-0)	1.00	no

ID: patient ID, Clone ID corresponds to sub-clone ID in Supplementary Figures 3-26.
* Contribution (95% confidence interval) of the single-base substitution (SBS)-MM1 signature according to mmsig.
** Presence of SBS-MM1 was further checked using mSigAct (likelihood ratio test). P values were not corrected for multiple testing.
*** yes: both tests (mmsig & mSigAct) indicated presence of SBS-MM1

Supplementary Table 3: Clonal sweeps and best prior treatment response

ID	Sweep 1*	Prior response***	Sweep 2*	Prior response	Sweep 3*	Prior response
1	x**	CR	x	PR	x	PD
2	-		-		-	
6	x	VGPR	-		-	
7	x	CR	-		-	
8	x	PR	-		-	
12	x	VGPR	x	CR	x	PD
14	x	PR	x	PR	-	
15	x	CR	x	CR	-	
16	x	CR	x	PD	-	
17	x	CR	-		-	
19	x	CR	-		-	
20	x	CR	x	VGPR	-	
22	x	CR	x	PR	-	
24	x	CR	x	PD	-	

*Sweep 1-3: consecutive sub-clonal sweeps; **x: clonal sweep; ***Level of response during prior treatment; PR: partial response; VGPR: very good partial response; CR: complete response; PD: progressive disease

Supplementary Table 4: Mutations in epigentic modifiers of KDM/KMT family

ID	Gene	Chr	Pos	Ref	Alt	Mutation type	Protein change	Sub-clone[1]
3	KMT2B	19	36223964	G	T	missense	Val2172Phe	1.1
6	KDM2A	11	67010546	T	G	missense	Trp516Gly	1.1.2
7	KDM3A	2	86683616	C	T	missense	Ser203Phe	1.1
7	KDM3B	5	137760018	G	A	stop gained	Trp1409*	1.2.2.1
7	KDM5B	1	202700034	C	T	splice site	-	1.1
7	KDM5B	1	202727542	C	A	missense	Asp428Tyr	1.2.2.1
7	KMT2C	7	151833961	G	T	missense	Pro4955Thr	1.2.2.1
10	KDM2A	11	66982864	C	A	missense	Ser180Arg	1.2.1
12	KDM4B	19	5131419	G	A	missense	Ala584Thr	1.1.1
12	KMT2D	12	49424479	C	G	missense	Gly4582Arg	1.1.3
14	KMT2C	7	151853012	G	C	missense	Asn4038Lys	1.1.1.1.1
15	KDM4C	9	6793039	G	C	missense	Met17Ile	1.2.1
15	KMT2D	12	49432602	C	T	missense	Gly2846Asp	1.1
18	KDM5B	1	202700030	C	T	splice site	-	1.2.2.1
20	KMT2D	12	49424767	T	G	missense	Lys4527Thr	1.1.2.2.1
24	KDM3B	5	137715291	G	T	missense	Gly200Val	2.1

ID: patient ID; [1]please refer to Supplementary Figures 3-26.

Supplementary Table 5: Sample inclusion

	Baseline comparison		Relapse comparison			Clonal relationship			
**ID**	**Iliac/Iliac**	**Iliac/FL**	**Iliac/Iliac**	**Iliac/FL or FL/FL**	**First/last sample**	**Paired Iliac/FL**	**Only Iliac**	**Evolution pattern**	**Sweeps**
1			x	x	x		x	x	x
2				x	x		x	x	x
3	x				x		x		
4		x			x	x	x		
5	x	x			x	x	x		
6	x	x			x	x	x	x	x
7	x	x	x		x	x	x	x	x
8		x			x	x	x	x	x
9	x				x		x	x	
10					x	x	x	x	
11					x		x	x	
12	x		x		x		x	x	x
13					x	x	x	x	
14	x			x	x		x	x	x
15		x			x	x	x	x	x
16					x	x	x	x	x
17				x	x			x	x
18	x	x			x	x	x	x	
19				x	x		x	x	x
20		x			x	x	x	x	x
21					x		x	x	
22					x		x	x	x
23				x	x	x	x	x	
24	x				x		x	x	x

Supplementary Figures

Supplementary Fig. 1: The number of sub-clones per patient. Boxplots for the number of sub-clones at baseline (iliac crest and focal lesion sub-clones combined), relapse (all follow-up iliac crest and focal lesion samples combined), or during the entire course of the disease (baseline & relapse -> complete) for all patients (n=24) in this study. Sub-clones were defined based on SciClone clusters and presence of at least three mutations or at least one copy number aberration. The boxplots show the median and the interquartile range, while the upper and lower whiskers show the highest and lowest value (excluding outliers), respectively. Source data are provided as a Source Data file.

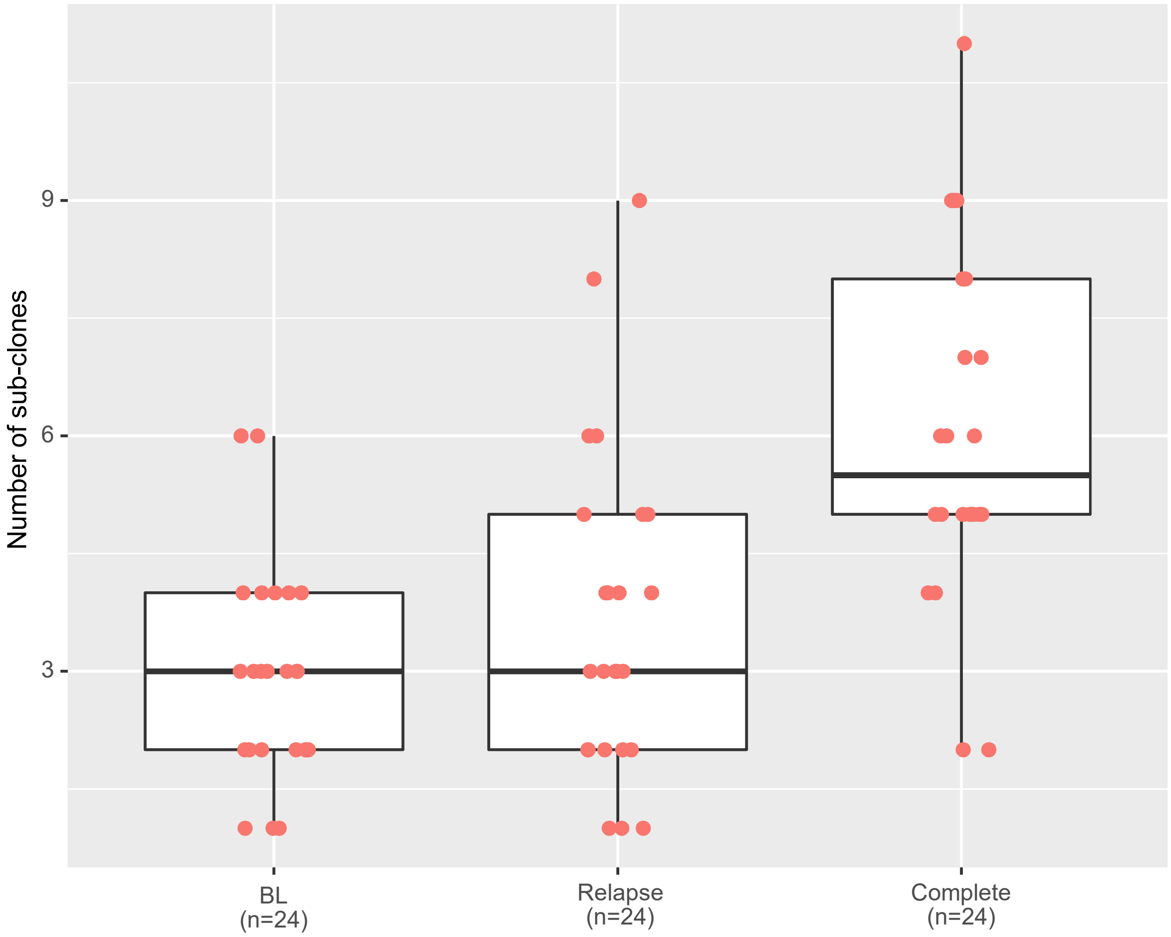

Supplementary Figure 2: Description of Supplementary Figures 3-27. The figure describes the cancer clonal fraction, mock ongogenetic tree and pie chart plots shown in Supplementary Figures 3-27. Clonal substructures were inferred using SciClone with the filtered set of SNVs and default parameters, except for minimumDepth, which was set to 50. For the manual design of mock phylogenetic trees, the output of SciClone was further interpreted after inclusion of copy number data.

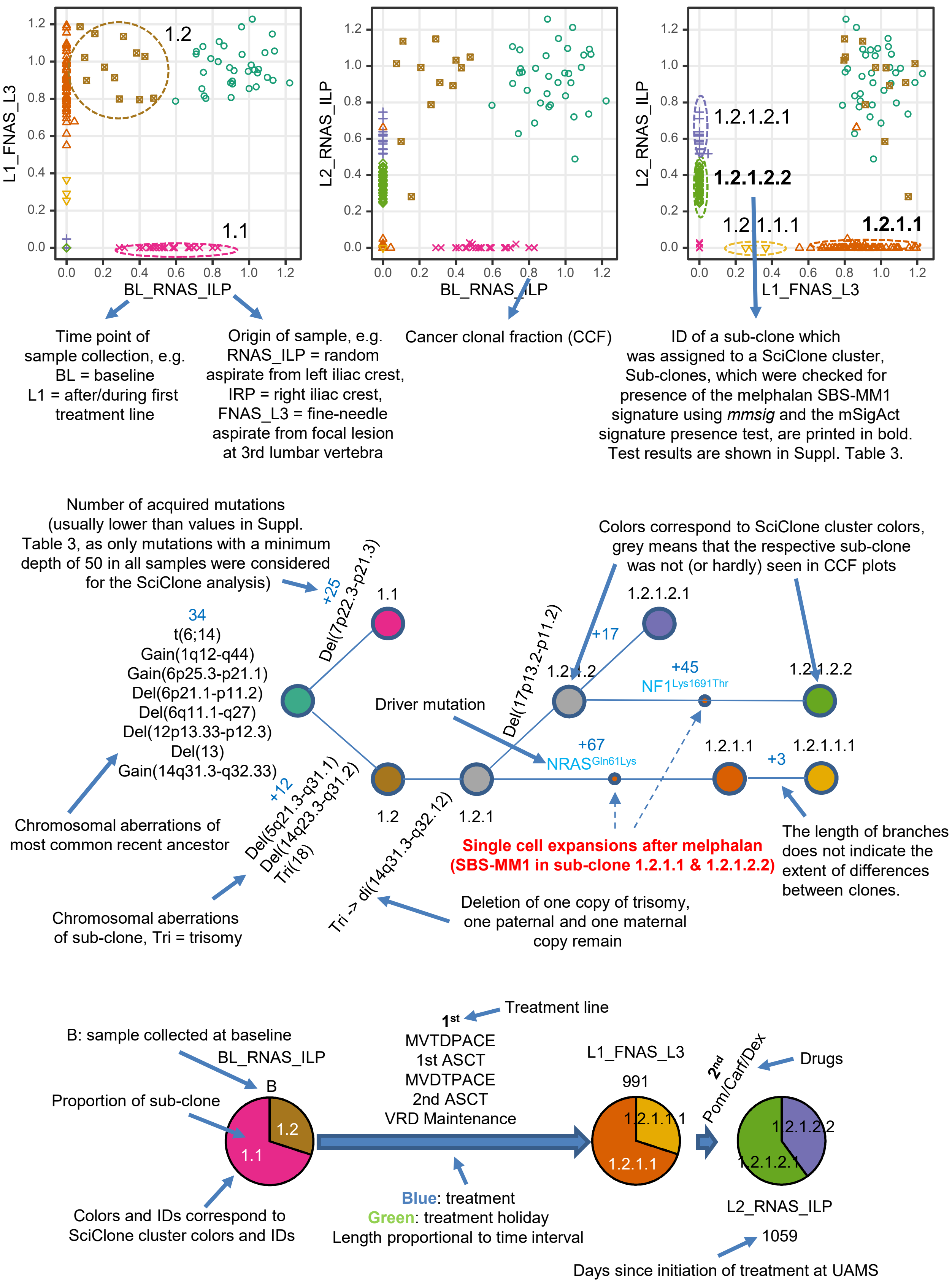

Supplementary Figure 3: Cancer clonal fraction plots, oncogenetic tree, and pie charts for the sub-clonal composition alongside treatment information for patient #1. A detailed legend is provided in Suppl. Fig. 2. Source data are provided as a Source Data file.

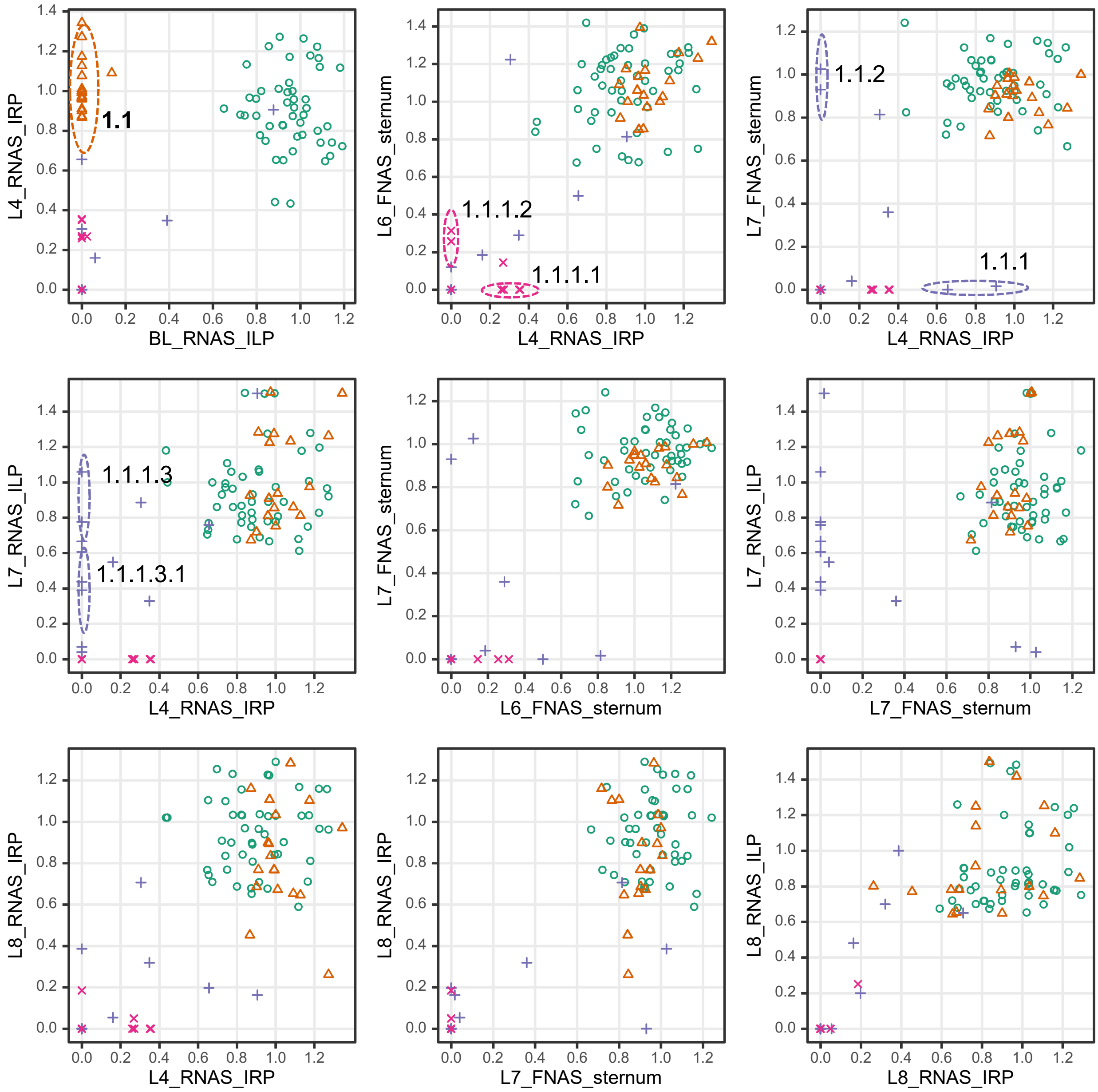

Supplementary Figure 3 (continued)

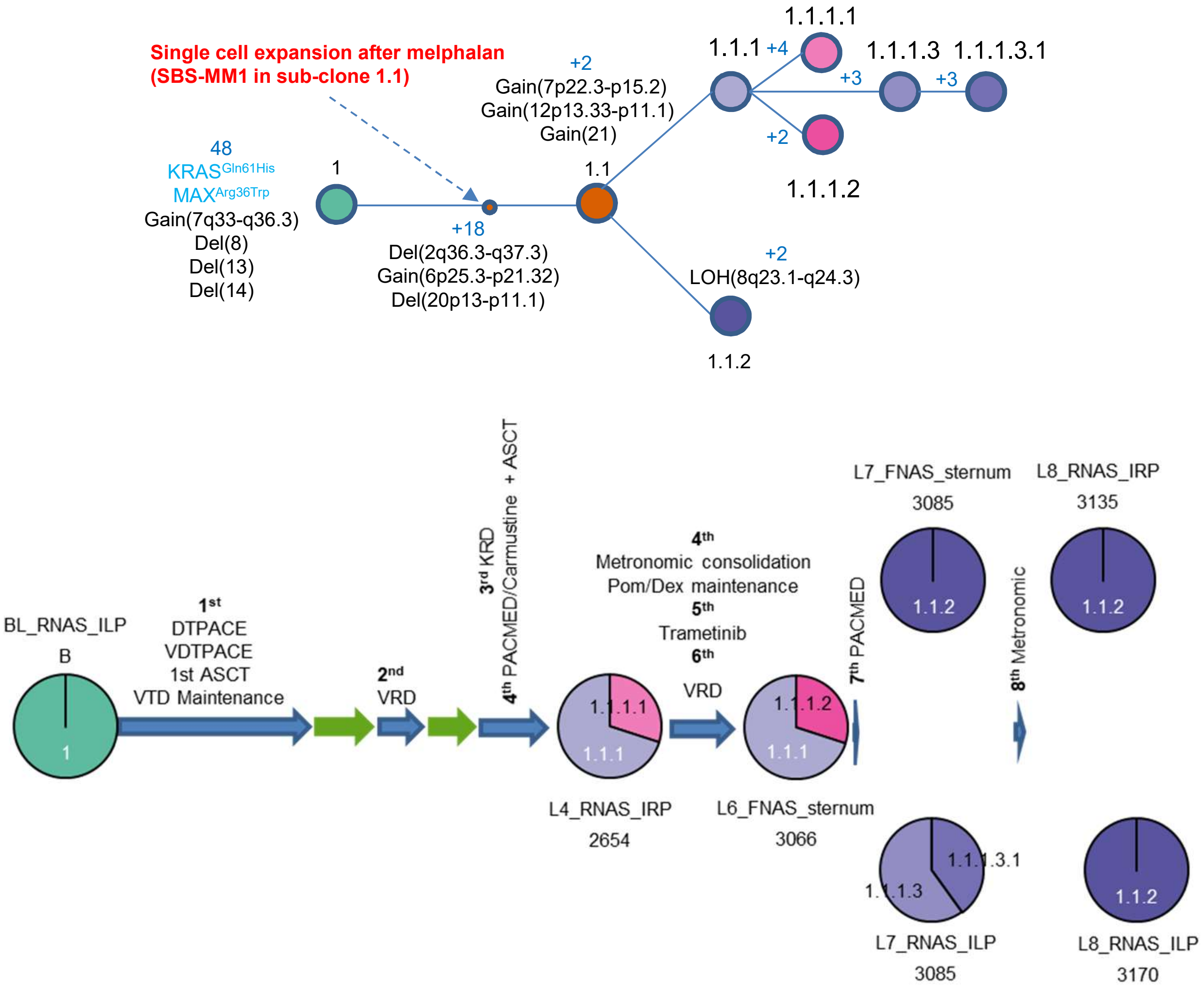

Supplementary Figure 4: Cancer clonal fraction plots, oncogenetic tree, and pie charts for the sub-clonal composition alongside treatment information for patient #2. A detailed legend is provided in Suppl. Fig. 2. Source data are provided as a Source Data file.

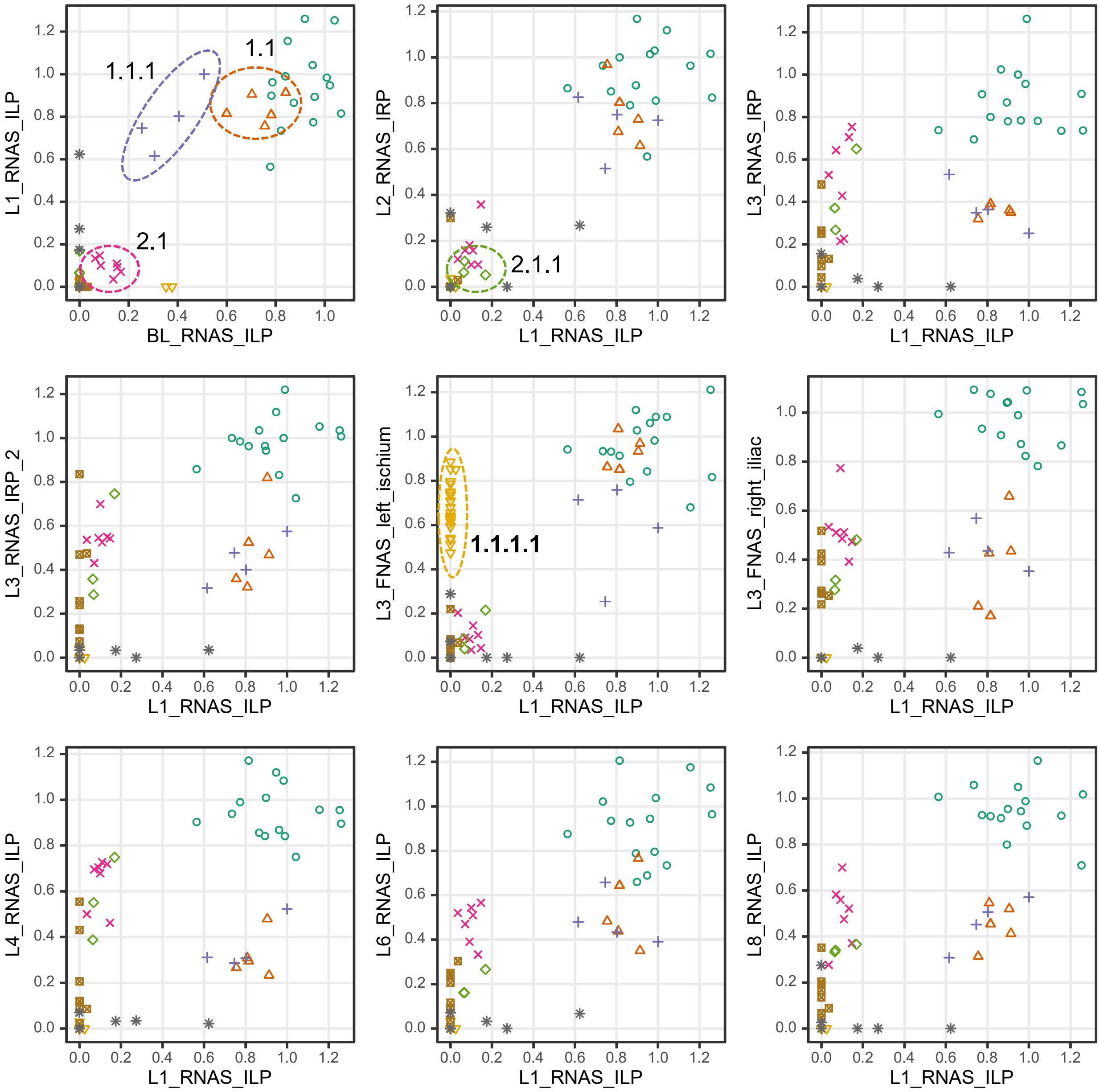

Supplementary Figure 4 (continued)

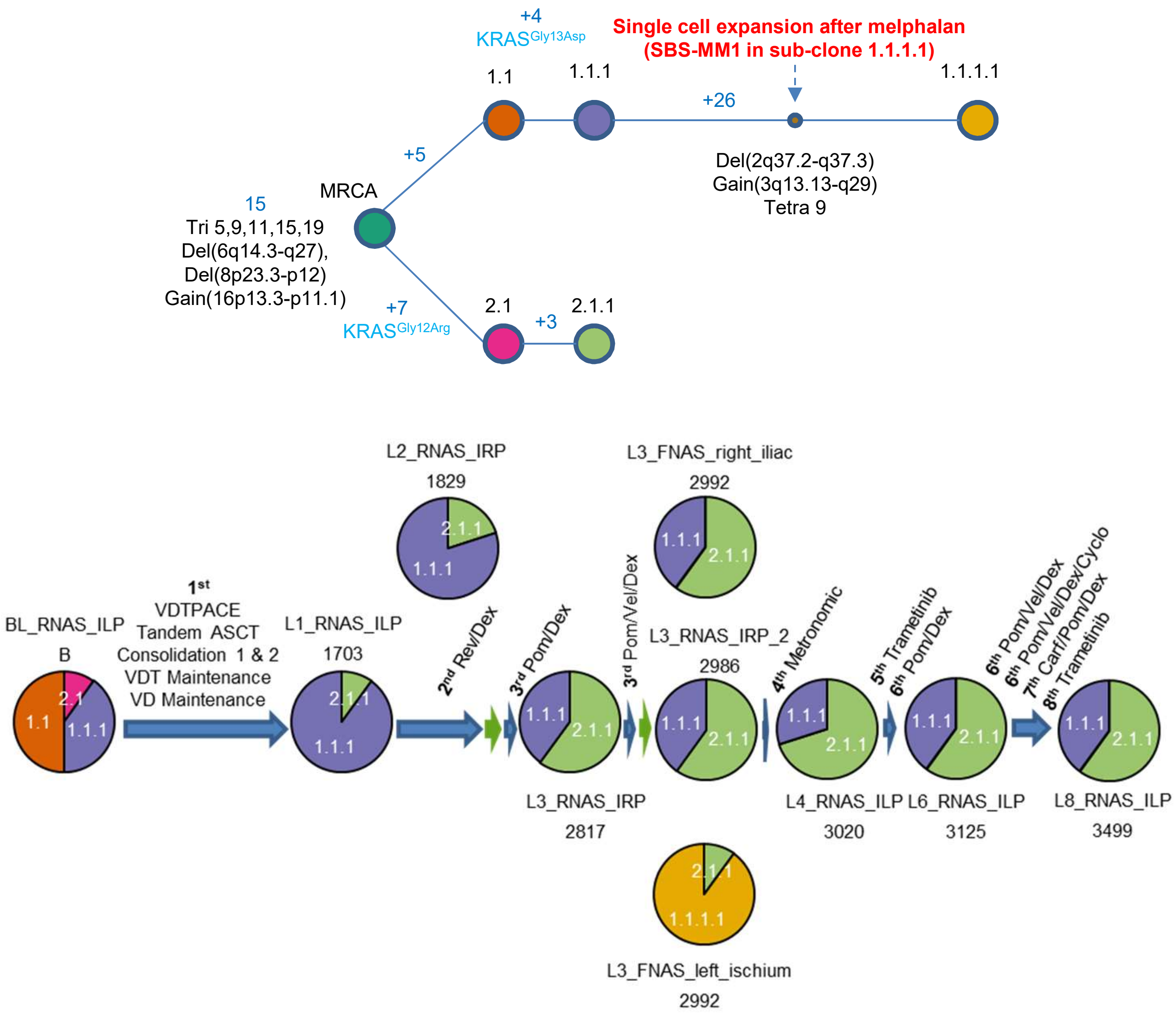

Supplementary Figure 5: Cancer clonal fraction plots, oncogenetic tree, and pie charts for the sub-clonal composition alongside treatment information for patient #3. A detailed legend is provided in Suppl. Fig. 2. Source data are provided as a Source Data file.

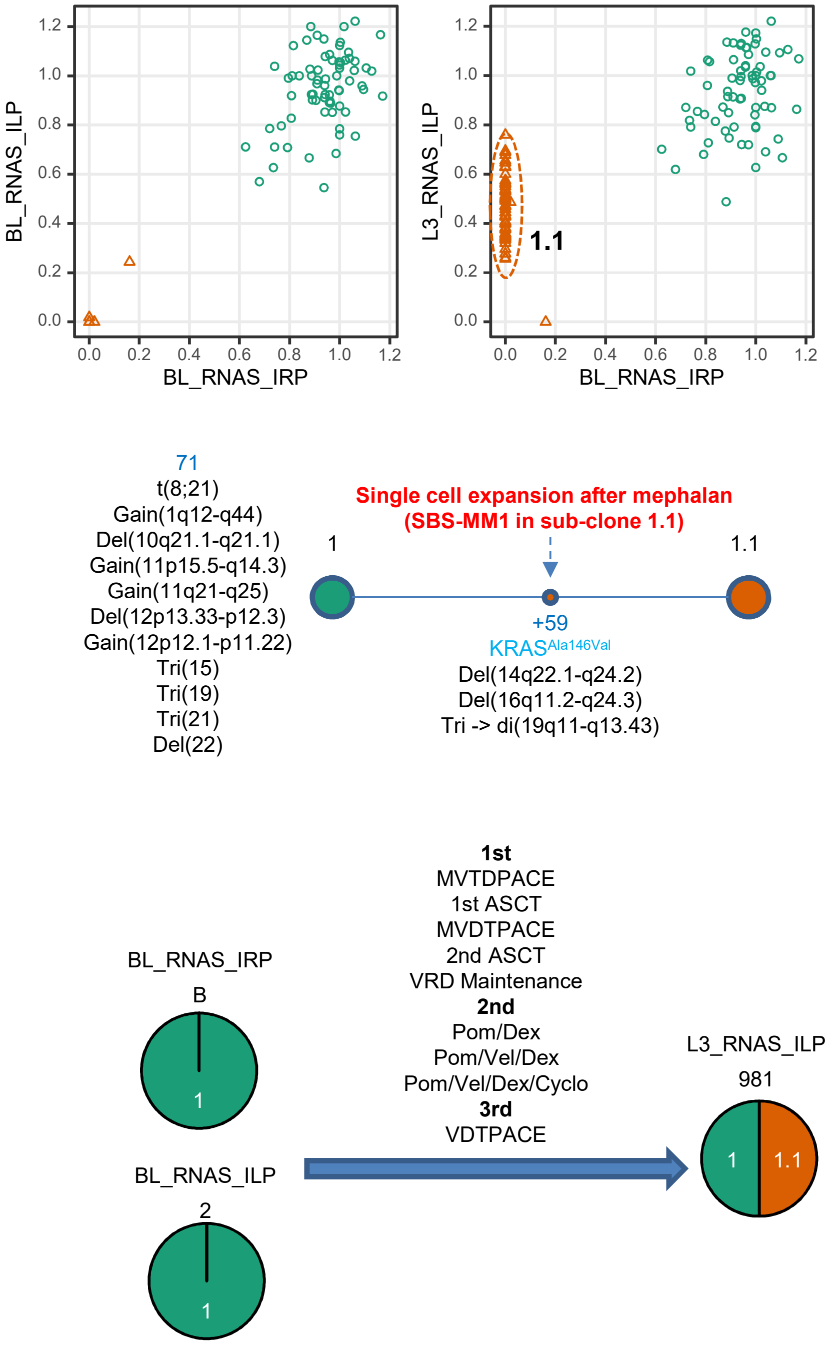

Supplementary Figure 6: Cancer clonal fraction plots, oncogenetic tree, and pie charts for the sub-clonal composition alongside treatment information for patient #4. A detailed legend is provided in Suppl. Fig. 2. Source data are provided as a Source Data file.

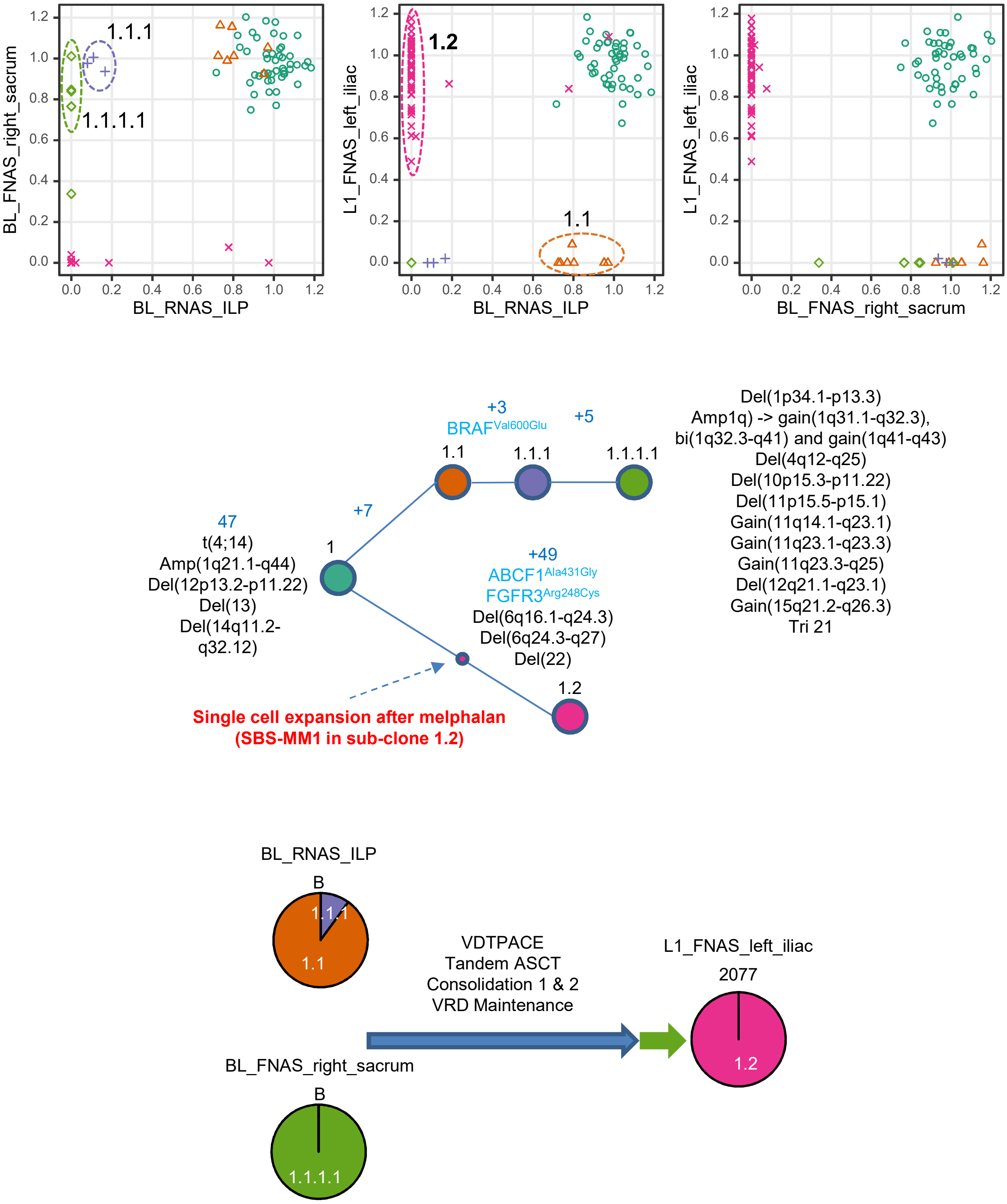

Supplementary Figure 7: Cancer clonal fraction plots, oncogenetic tree, and pie charts for the sub-clonal composition alongside treatment information for patient #5. A detailed legend is provided in Suppl. Fig. 2. Source data are provided as a Source Data file.

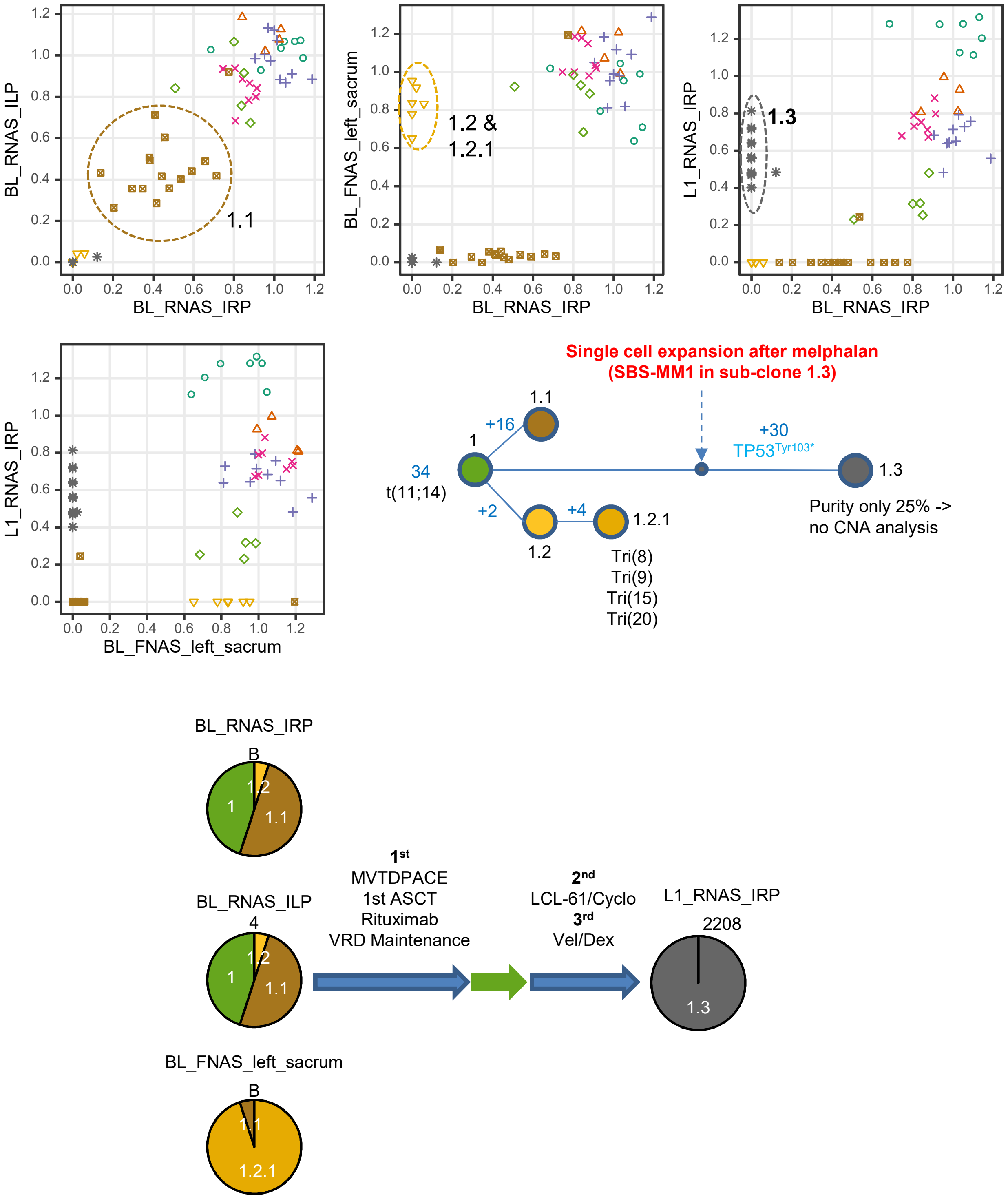

Supplementary Figure 8: Cancer clonal fraction plots, oncogenetic tree, and pie charts for the sub-clonal composition alongside treatment information for patient #6. A detailed legend is provided in Suppl. Fig. 2. Source data are provided as a Source Data file.

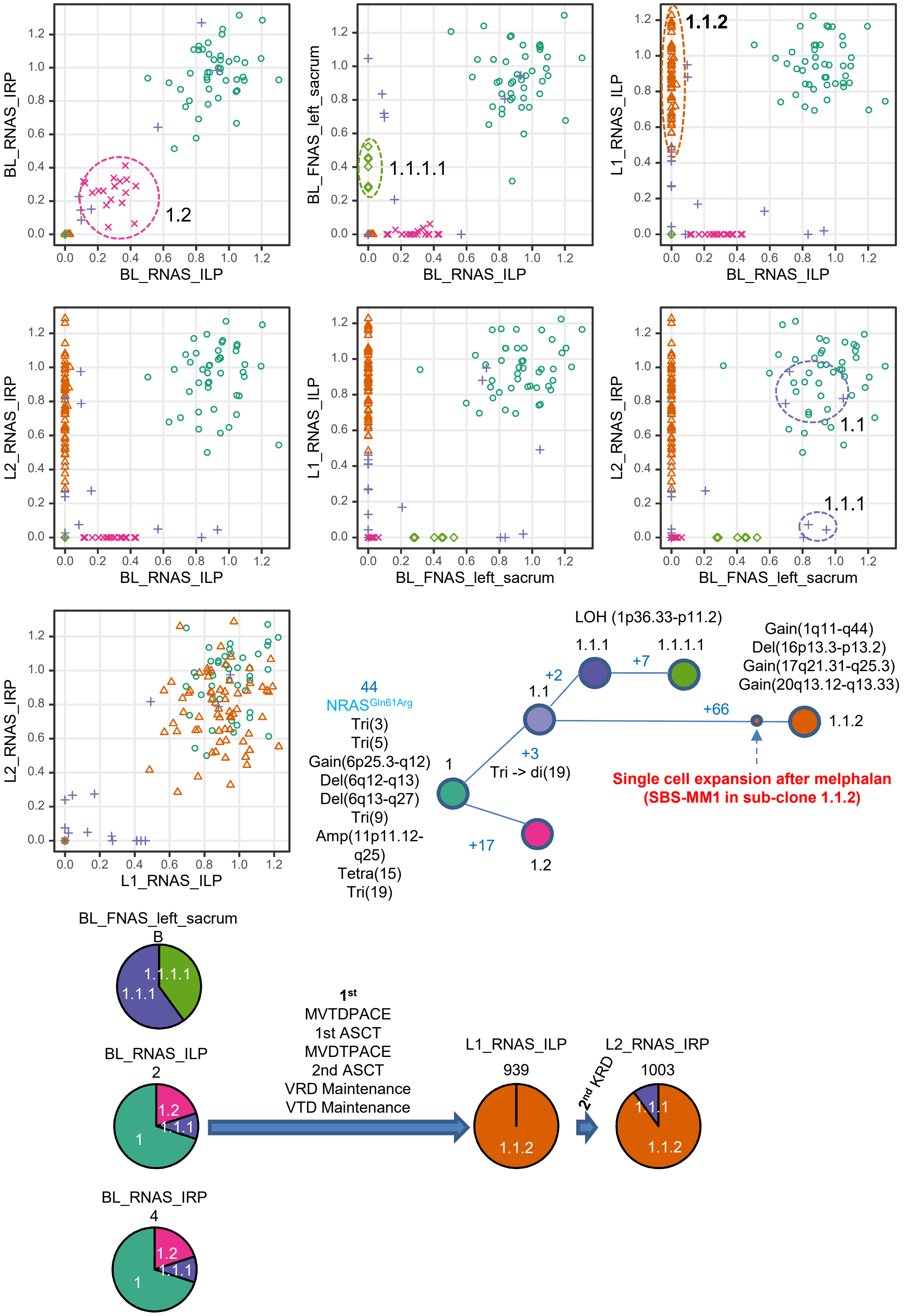

Supplementary Figure 9: Cancer clonal fraction plots, oncogenetic tree, and pie charts for the sub-clonal composition alongside treatment information for patient #7. A detailed legend is provided in Suppl. Fig. 2. Source data are provided as a Source Data file.

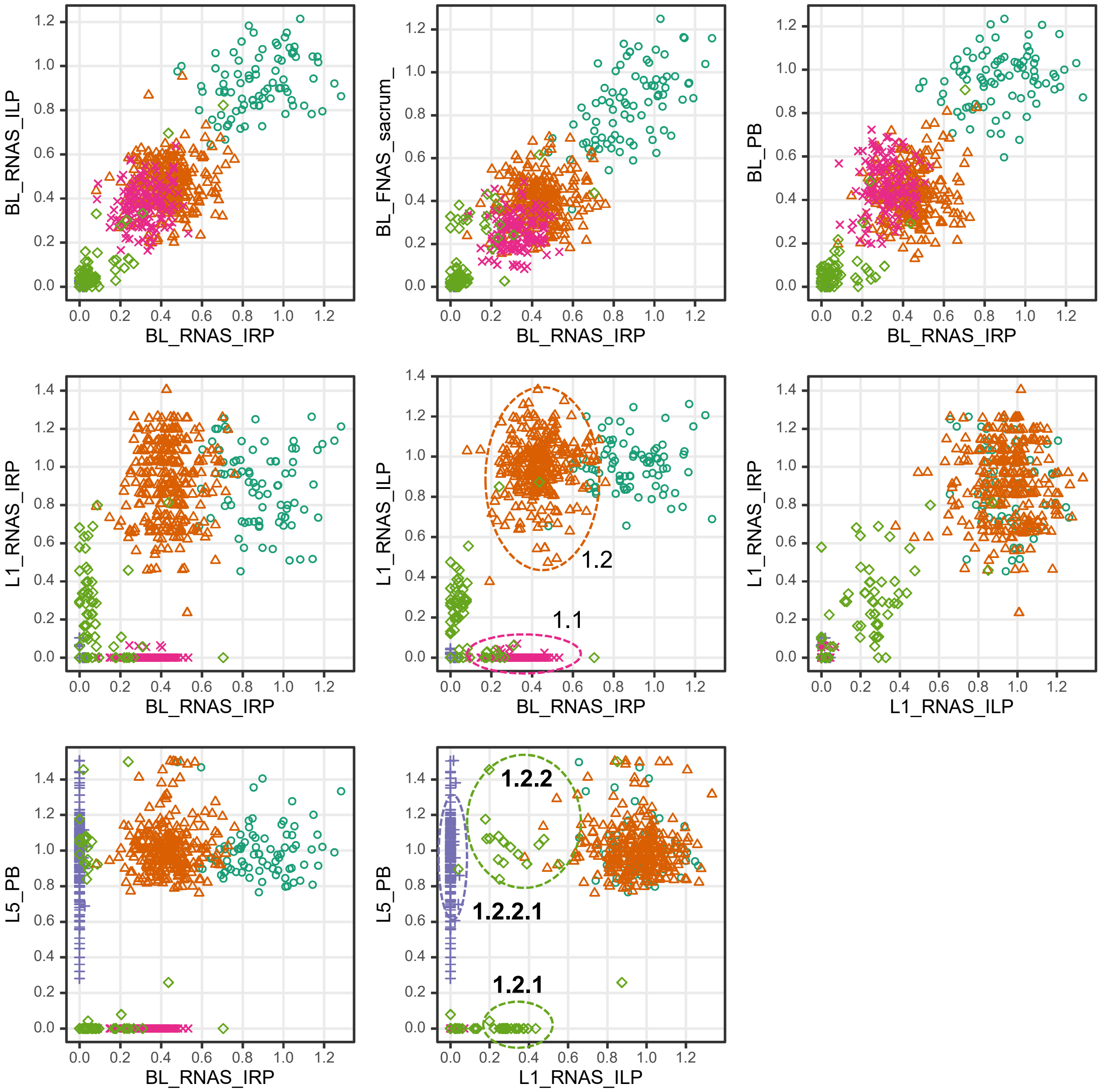

Supplementary Figure 9 (continued)

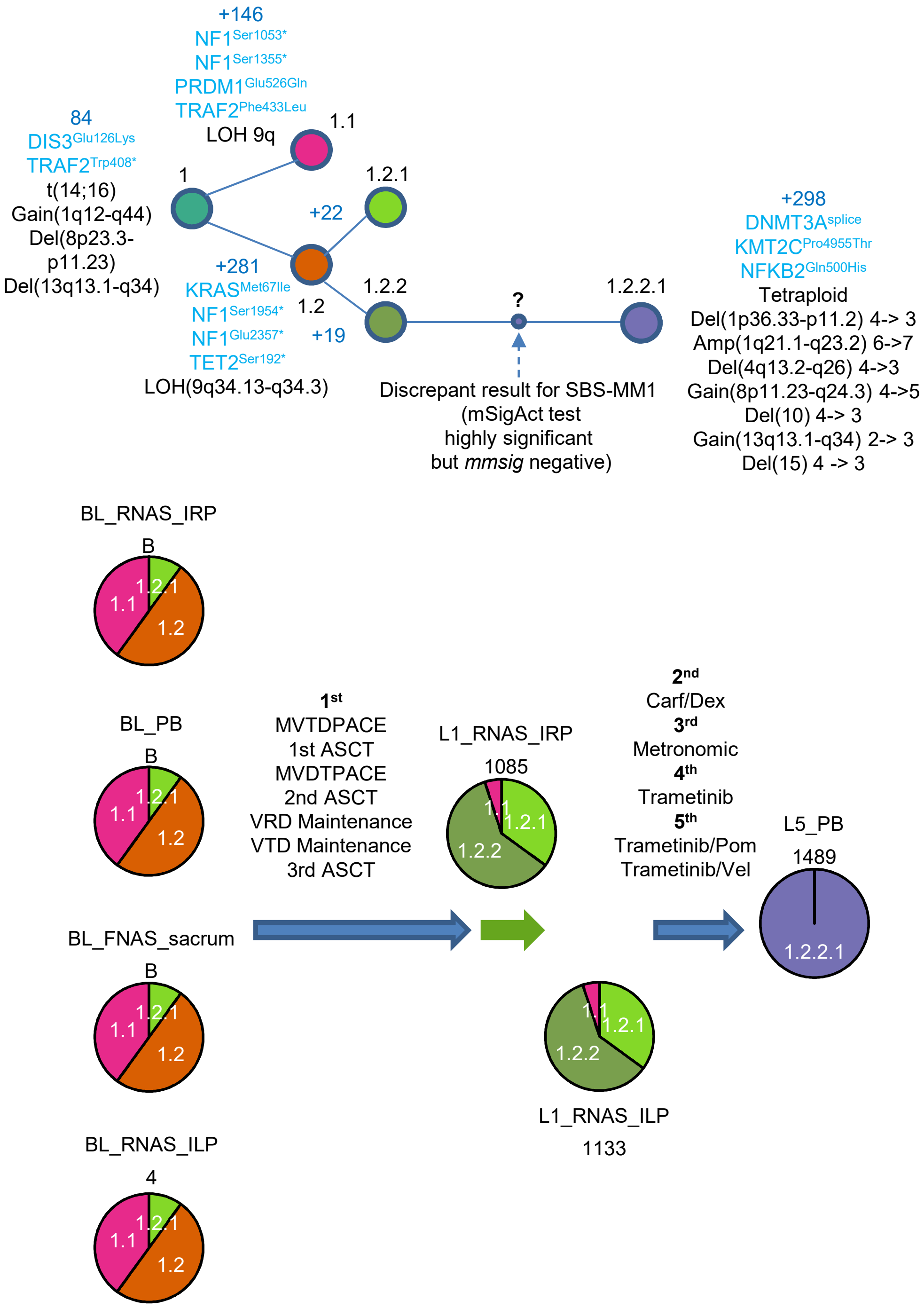

Supplementary Figure 10: Cancer clonal fraction plots, oncogenetic tree, and pie charts for the sub-clonal composition alongside treatment information for patient #8. A detailed legend is provided in Suppl. Fig. 2. Source data are provided as a Source Data file.

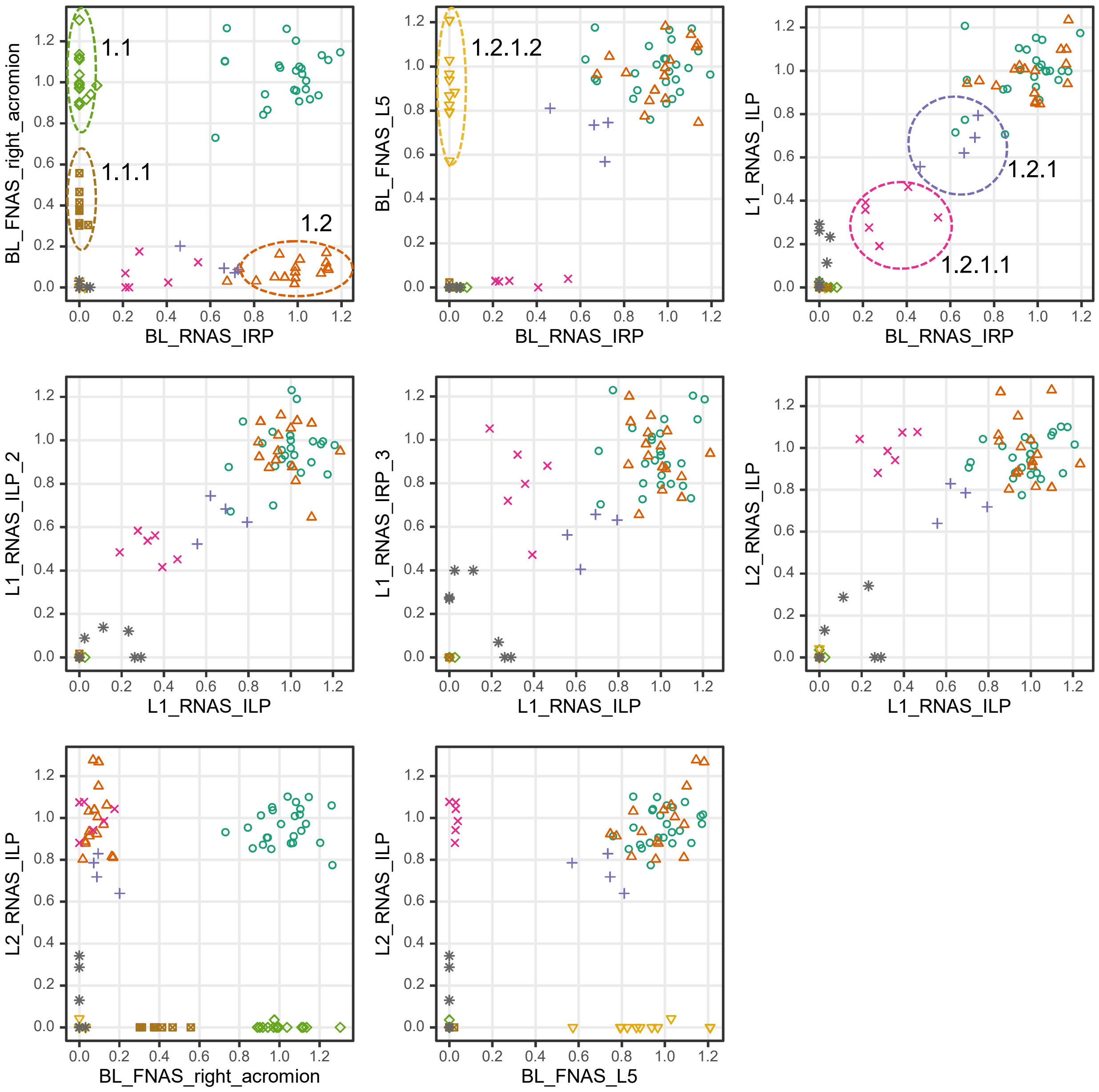

Supplementary Figure 10 (continued)

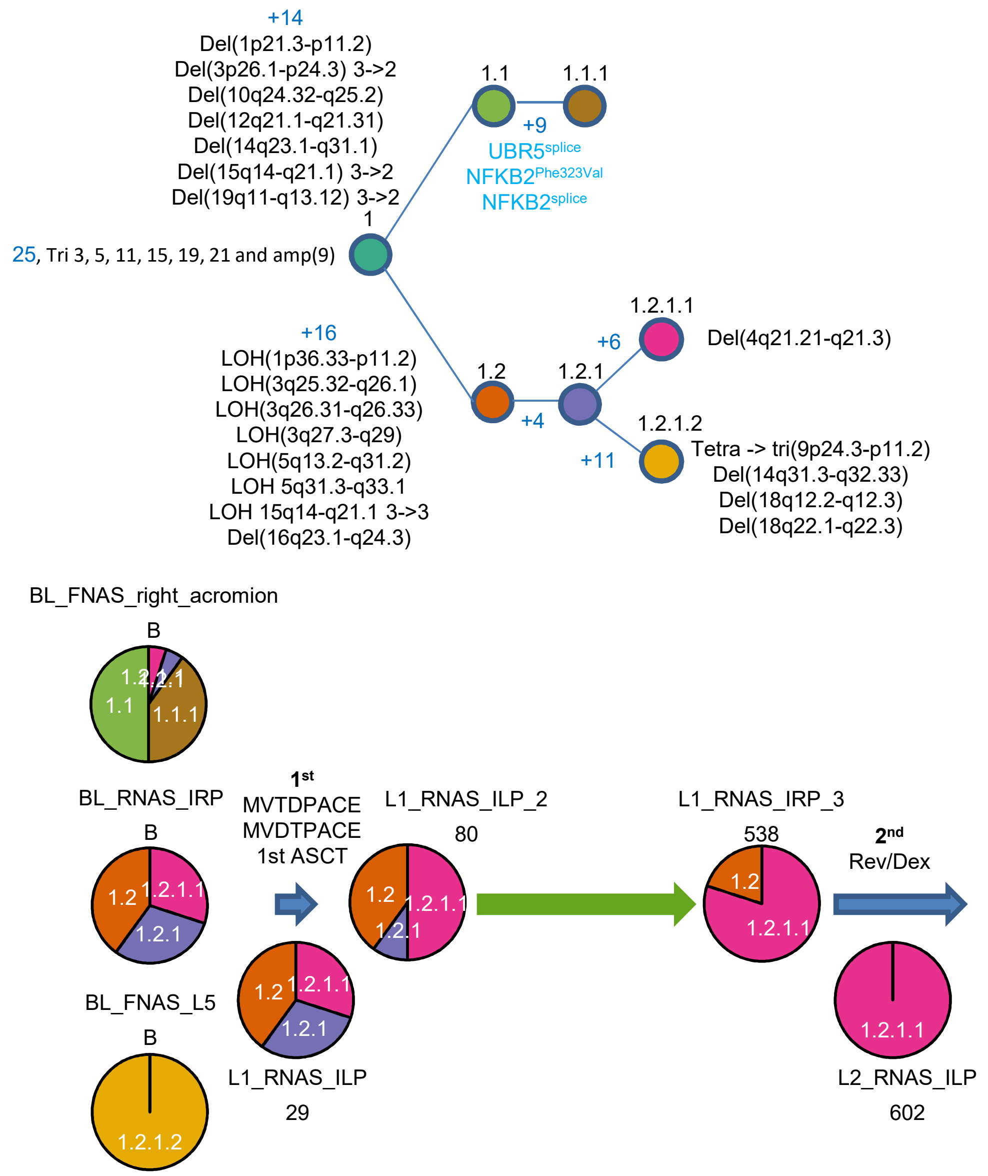

Supplementary Figure 11: Cancer clonal fraction plots, oncogenetic tree, and pie charts for the sub-clonal composition alongside treatment information for patient #9. A detailed legend is provided in Suppl. Fig. 2. Source data are provided as a Source Data file.

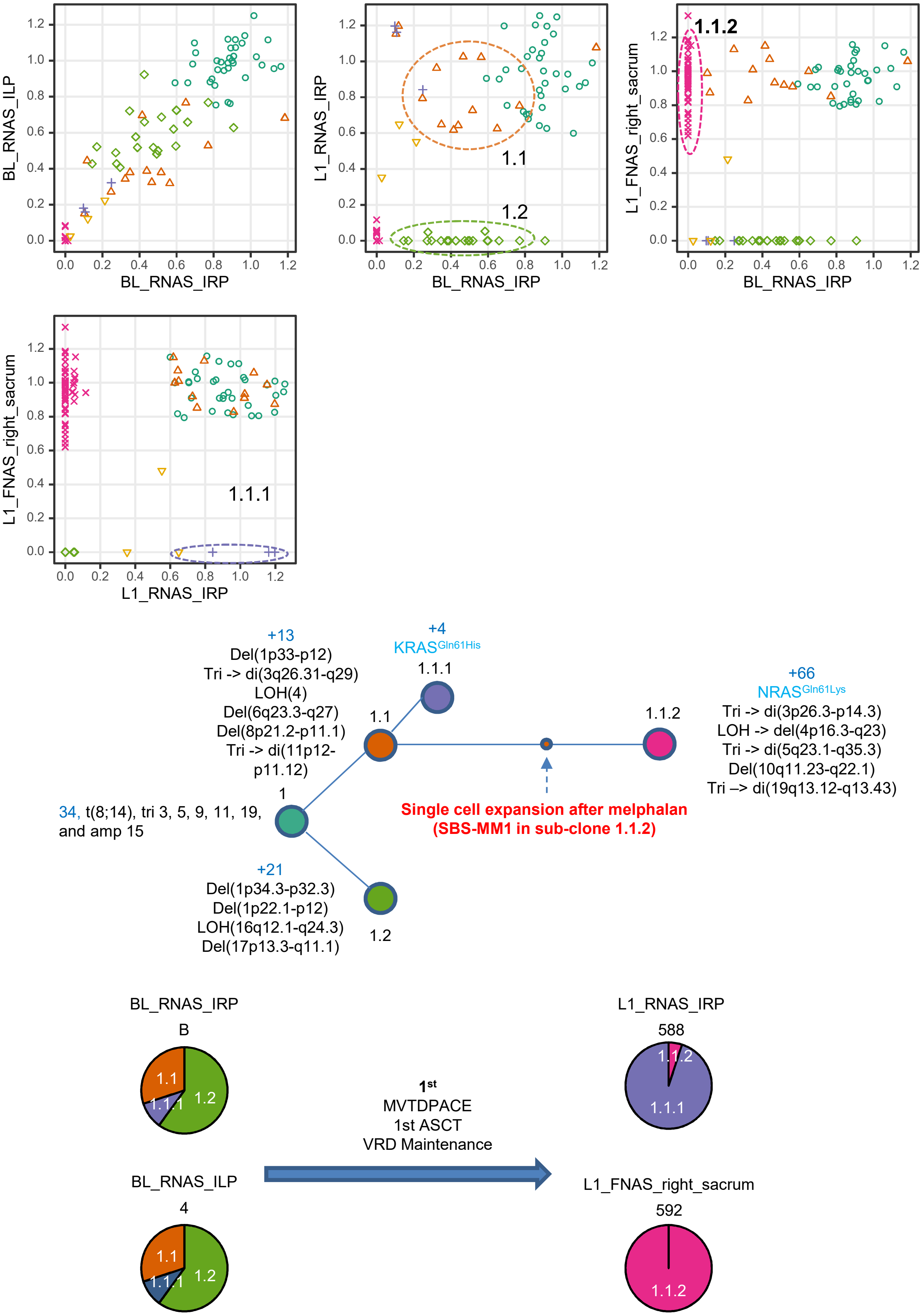

Supplementary Figure 12: Cancer clonal fraction plots, oncogenetic tree, and pie charts for the sub-clonal composition alongside treatment information for patient #10. A detailed legend is provided in Suppl. Fig. 2. Source data are provided as a Source Data file.

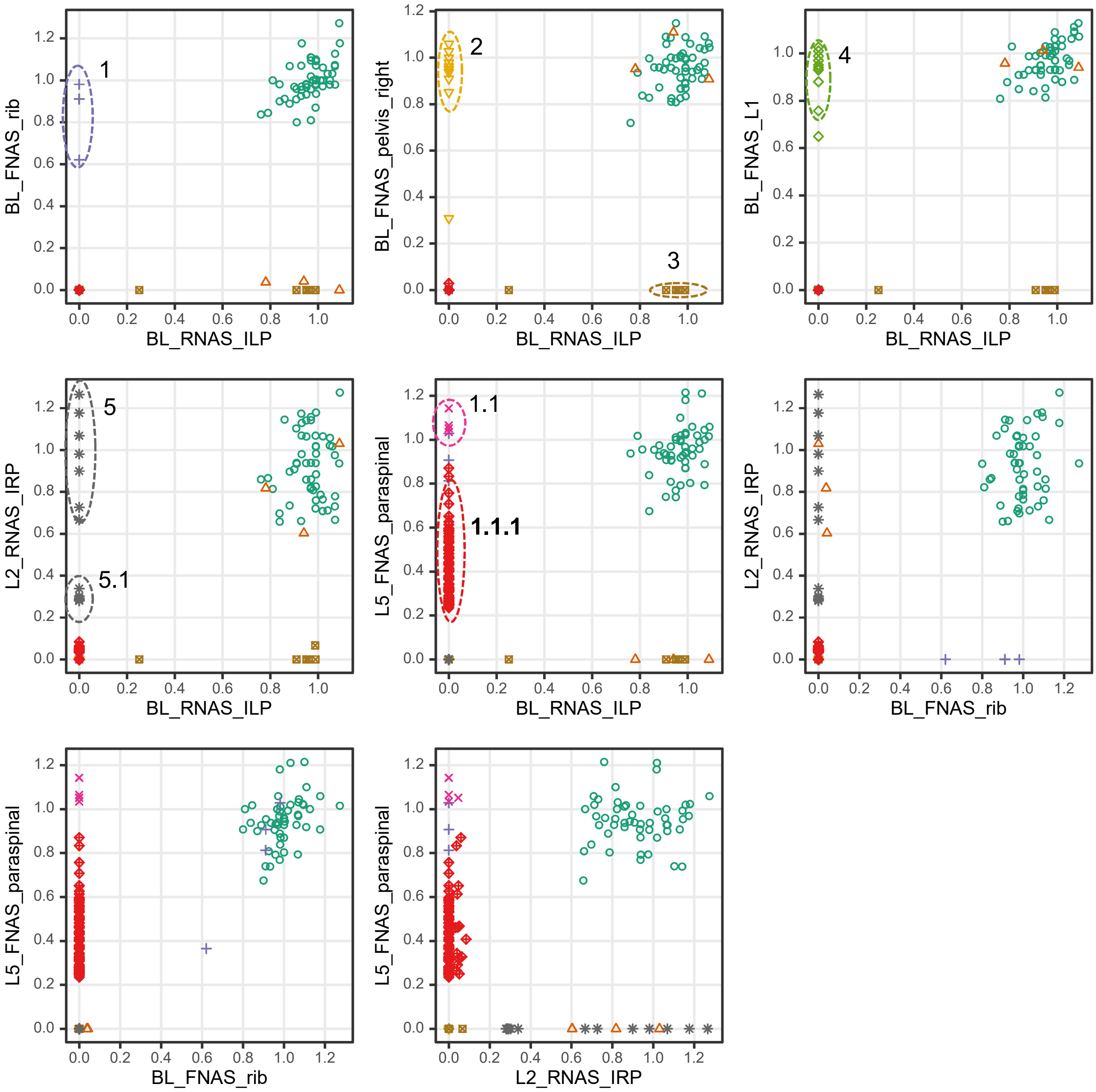

Supplementary Figure 12 (continued)

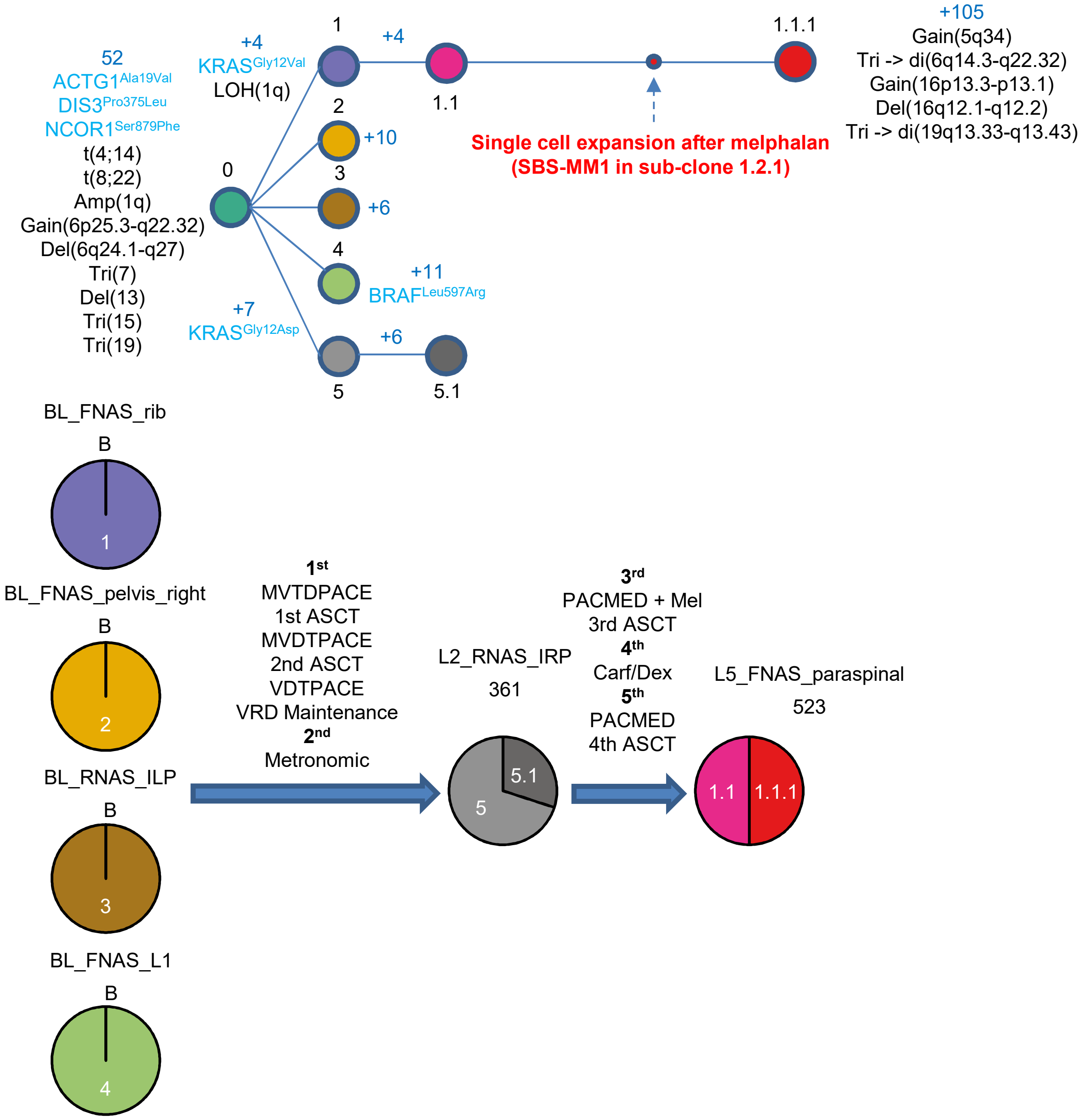

Supplementary Figure 13: Cancer clonal fraction plots, oncogenetic tree, and pie charts for the sub-clonal composition alongside treatment information for patient #11. A detailed legend is provided in Suppl. Fig. 2. Source data are provided as a Source Data file.

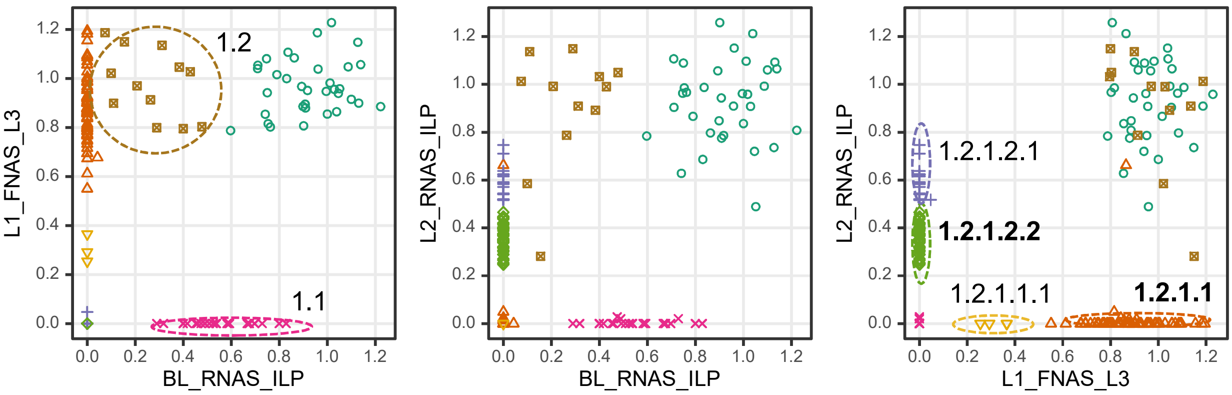

Supplementary Figure 13 (continued)

Solution A (green and lilac cluster represent two sub-clones with common ancestor (1.2.1.2))

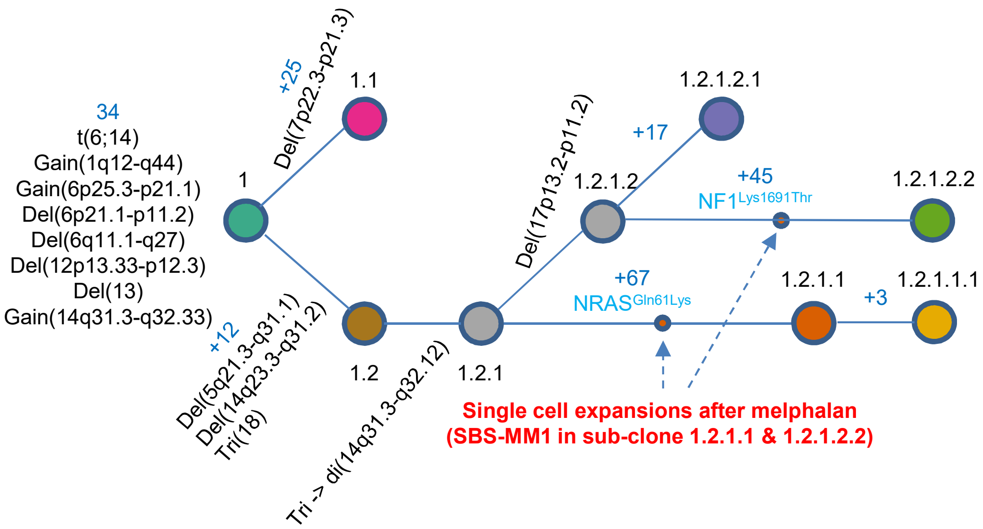

Solution B (green cluster represents descendant of lilac cluster)

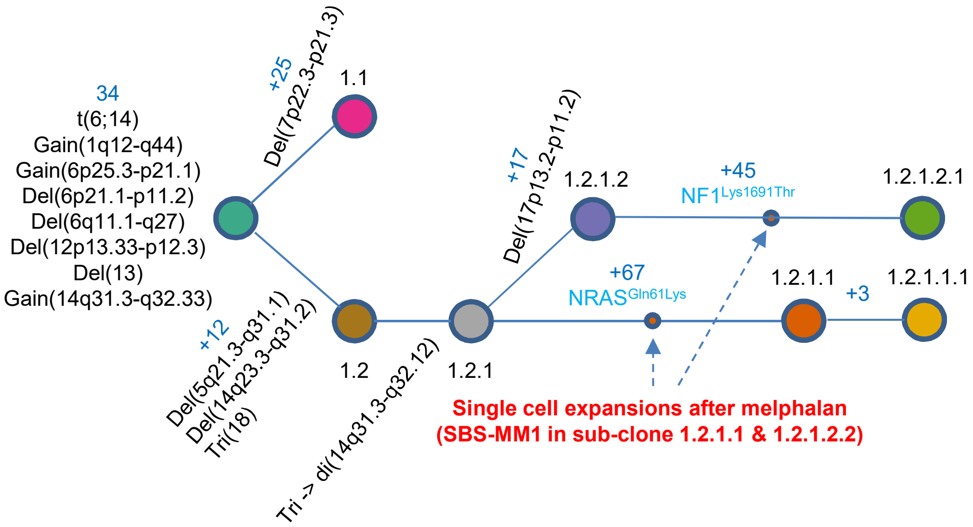

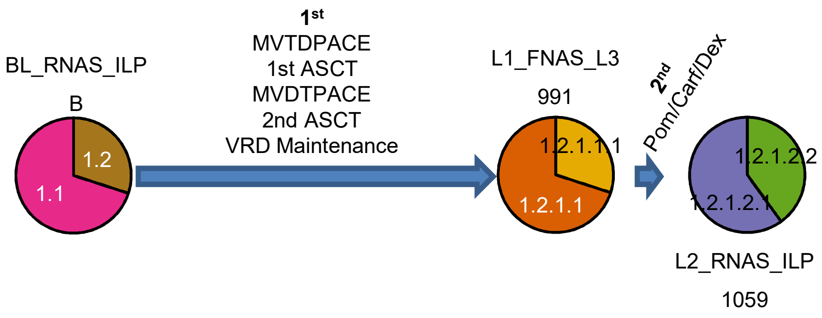

Supplementary Figure 14: Cancer clonal fraction plots, oncogenetic tree, and pie charts for the sub-clonal composition alongside treatment information for patient #12. A detailed legend is provided in Suppl. Fig. 2. Source data are provided as a Source Data file.

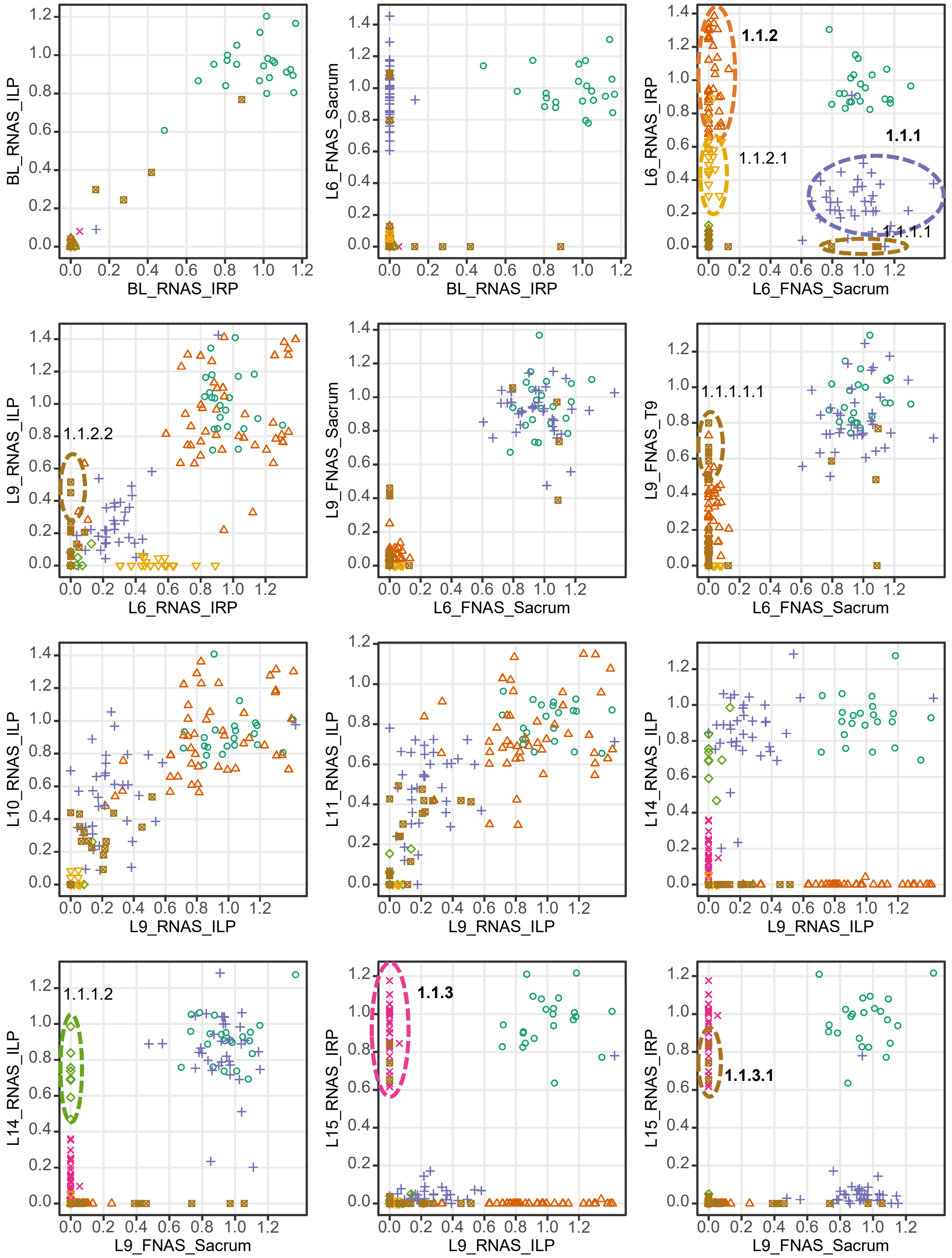

Supplementary Figure 14 (continued)

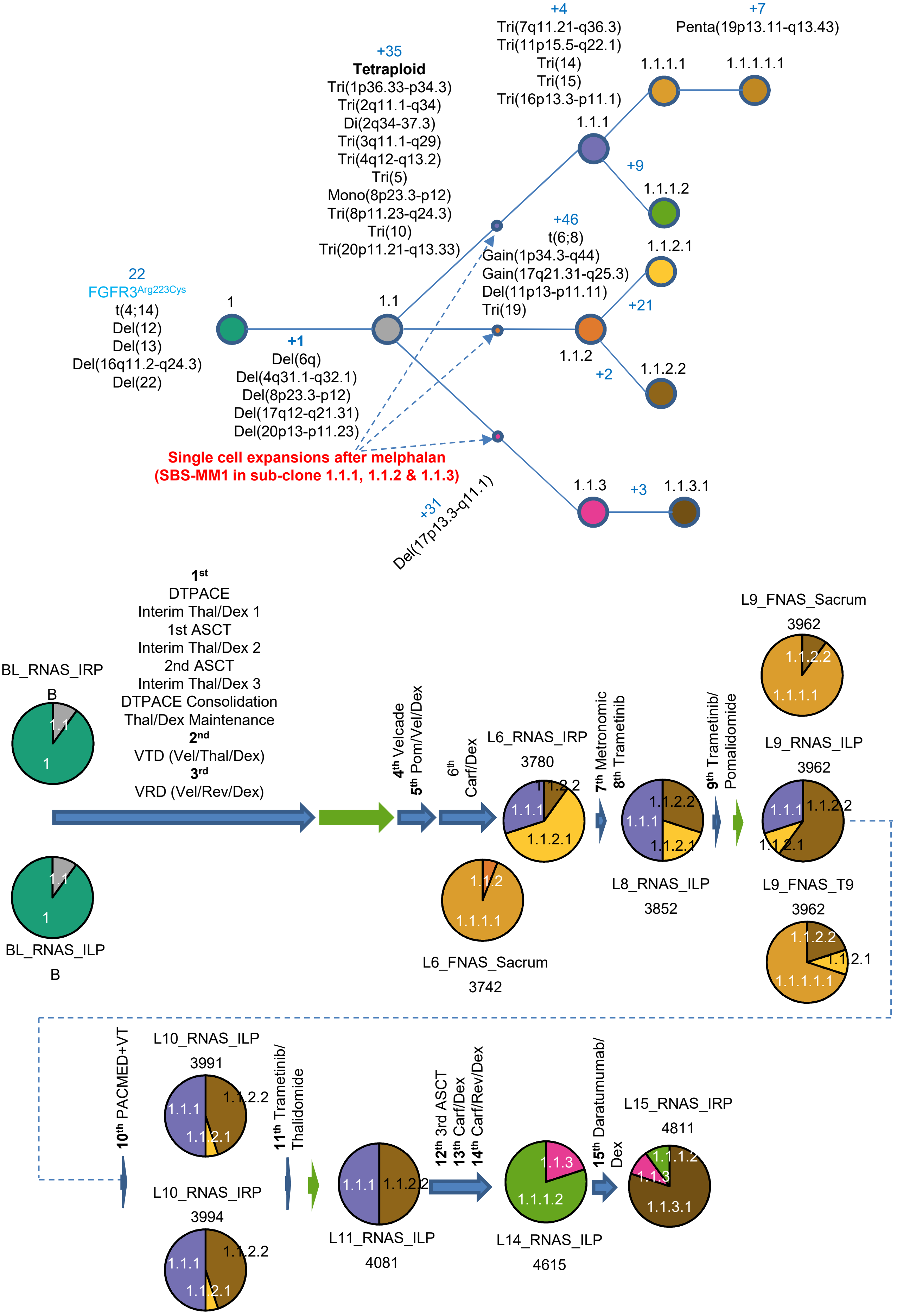

Supplementary Figure 15: Cancer clonal fraction plots, oncogenetic tree, and pie charts for the sub-clonal composition alongside treatment information for patient #13. A detailed legend is provided in Suppl. Fig. 2. Source data are provided as a Source Data file.

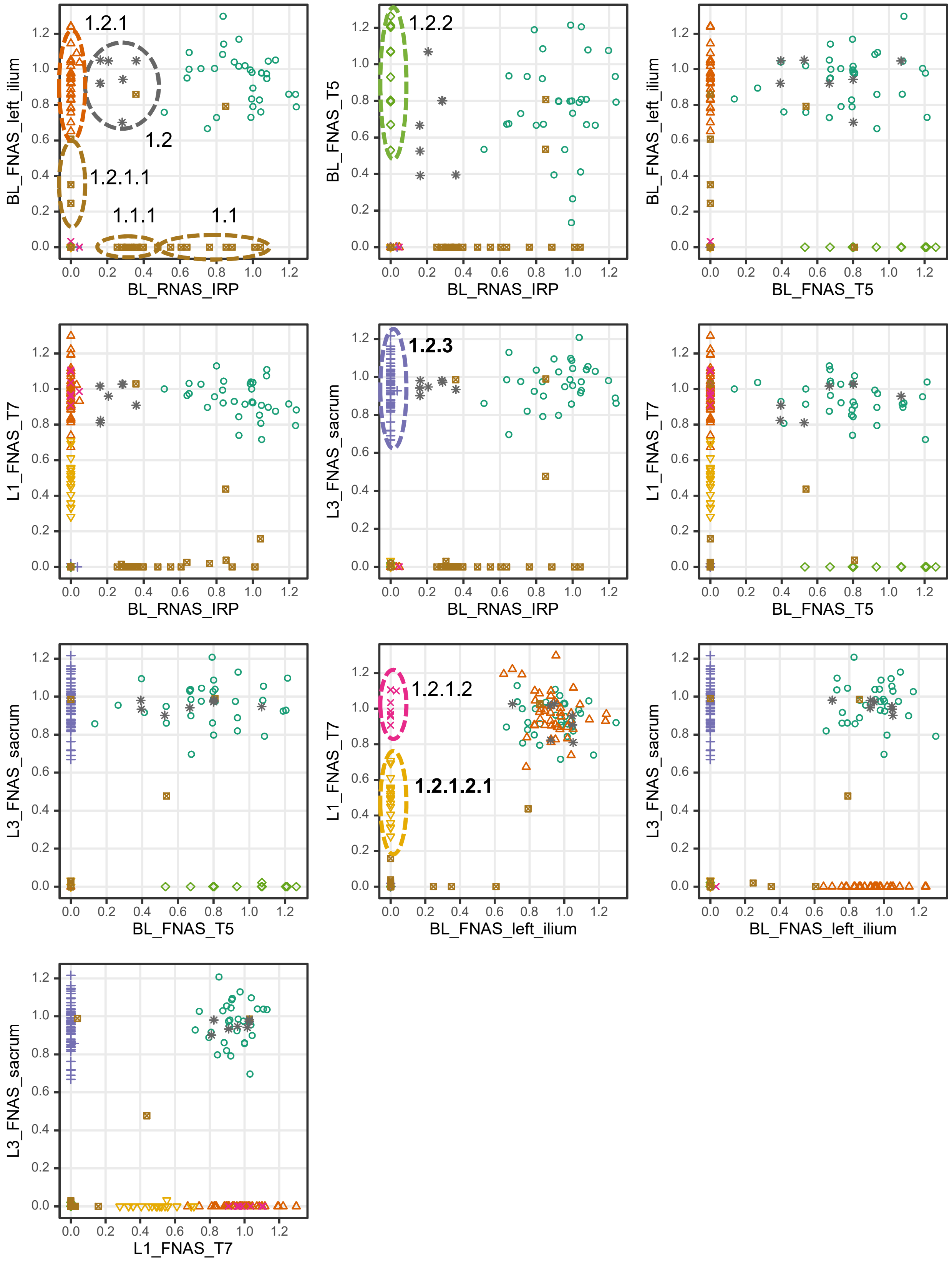

Supplementary Figure 15 (continued)

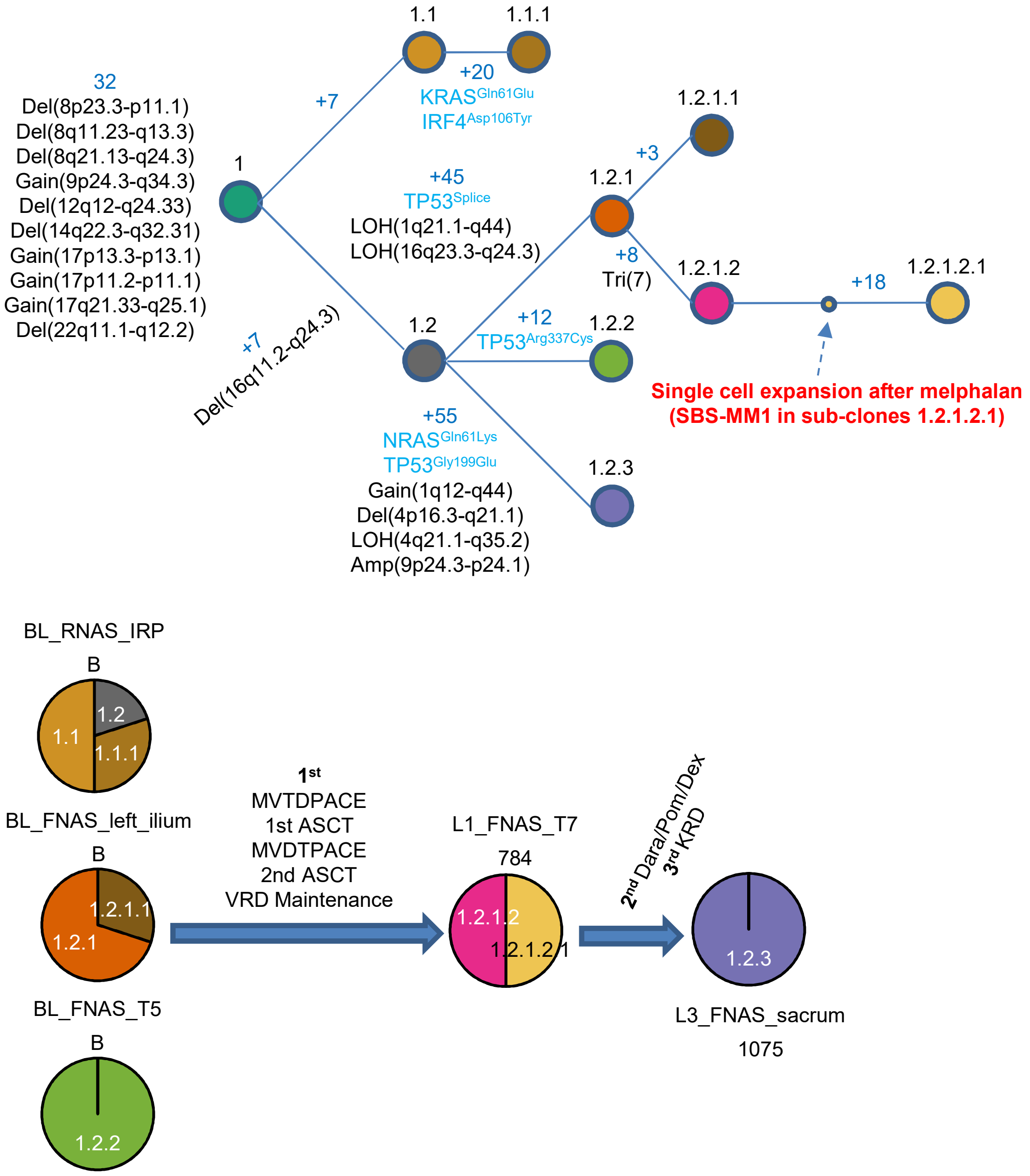

Supplementary Figure 16: Cancer clonal fraction plots, oncogenetic tree, and pie charts for the sub-clonal composition alongside treatment information for patient #14. A detailed legend is provided in Suppl. Fig. 2. Source data are provided as a Source Data file.

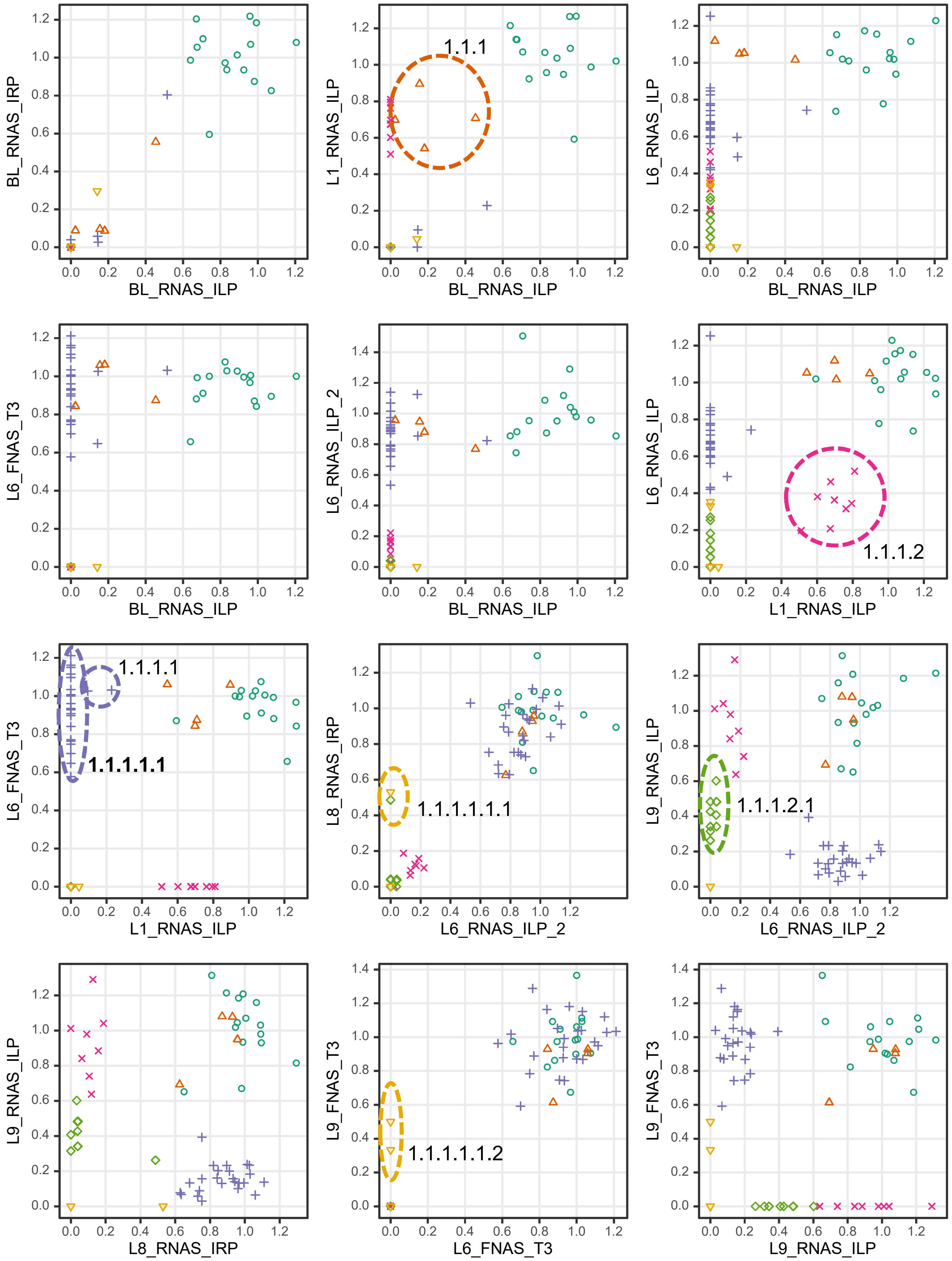

Supplementary Figure 16 (continued)

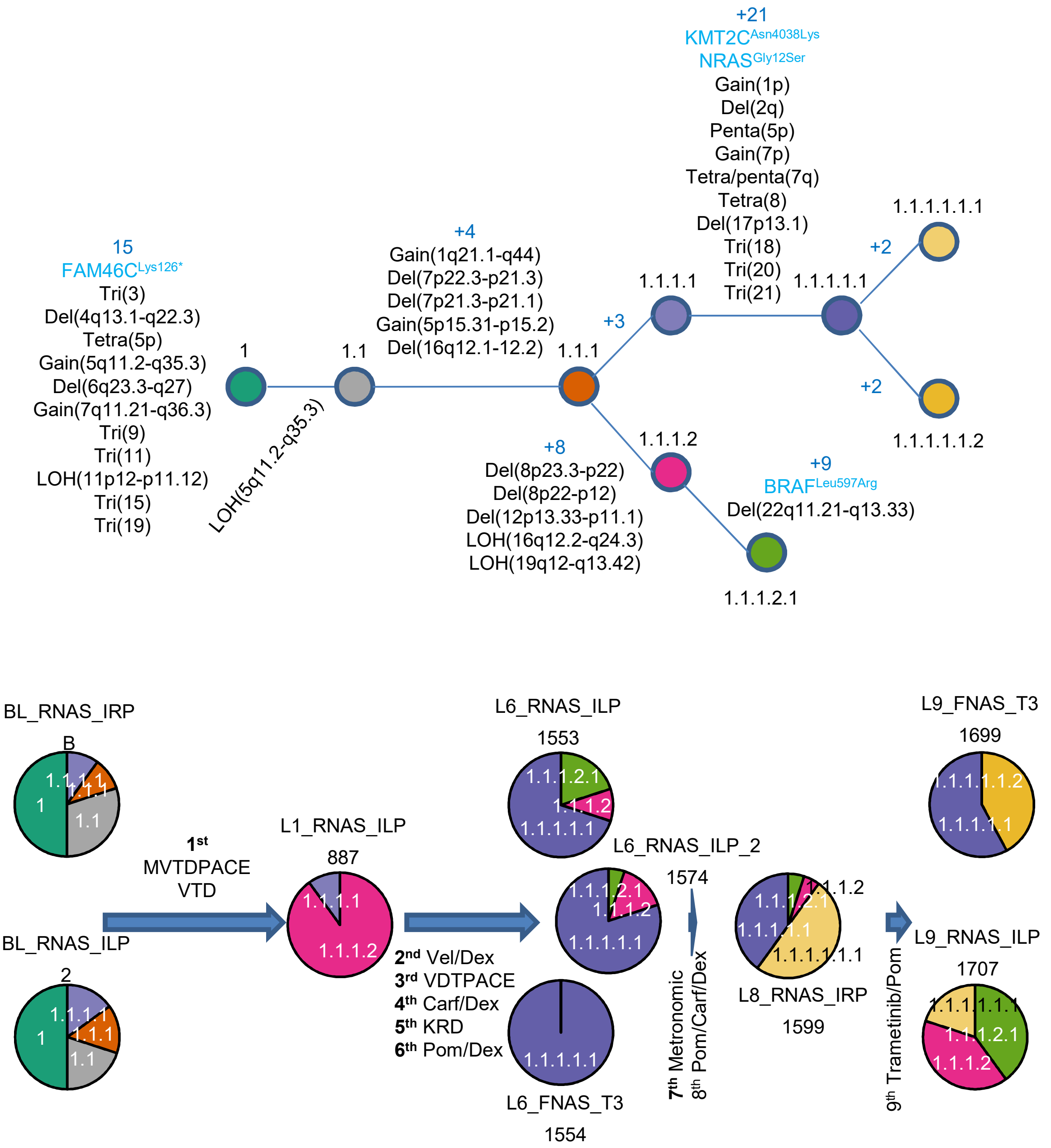

Supplementary Figure 17: Cancer clonal fraction plots, oncogenetic tree, and pie charts for the sub-clonal composition alongside treatment information for patient #15. A detailed legend is provided in Suppl. Fig. 2. Source data are provided as a Source Data file.

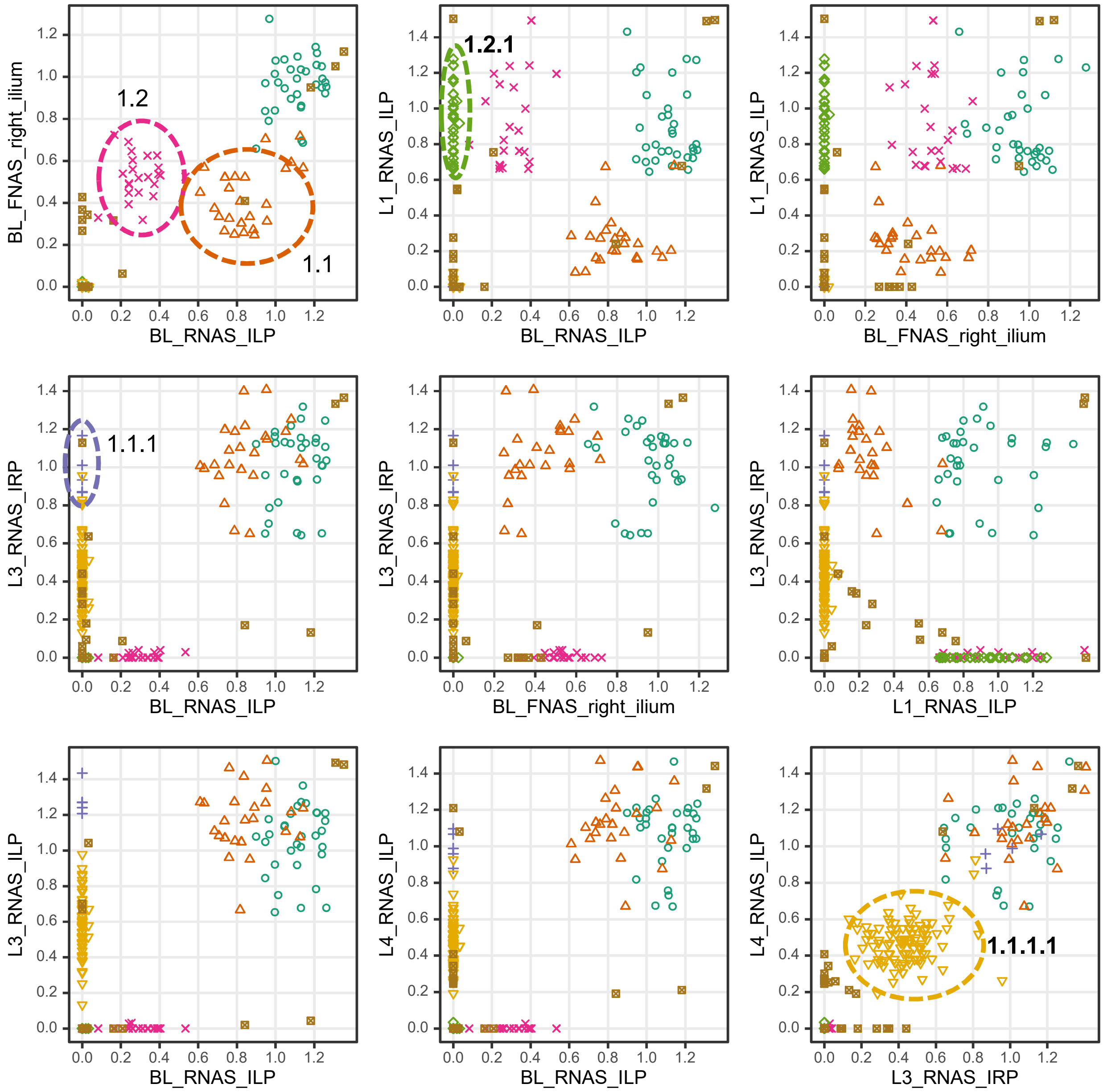

Supplementary Figure 17 (continued)

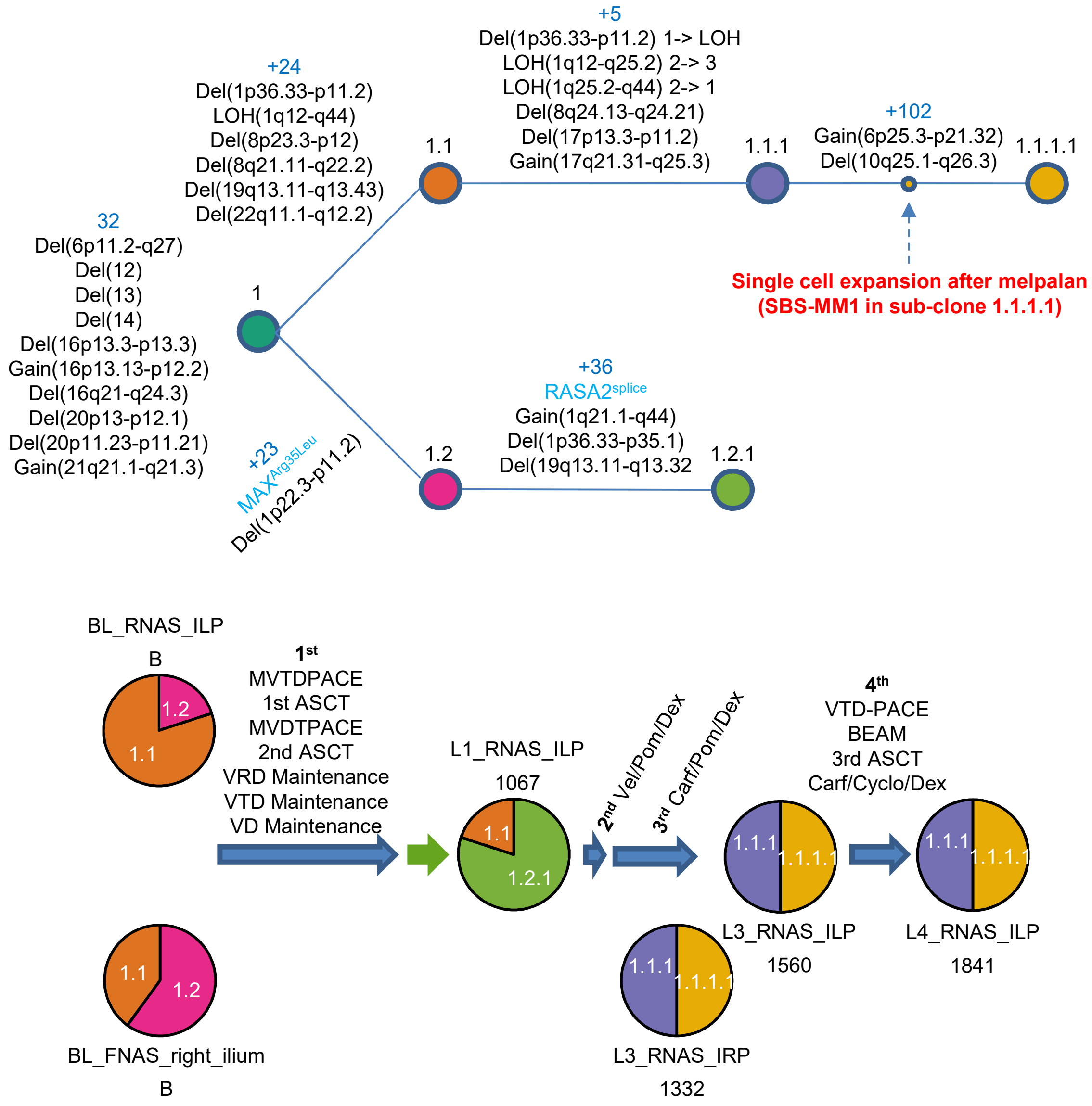

Supplementary Figure 18: Cancer clonal fraction plots, oncogenetic tree, and pie charts for the sub-clonal composition alongside treatment information for patient #16. A detailed legend is provided in Suppl. Fig. 2. Source data are provided as a Source Data file.

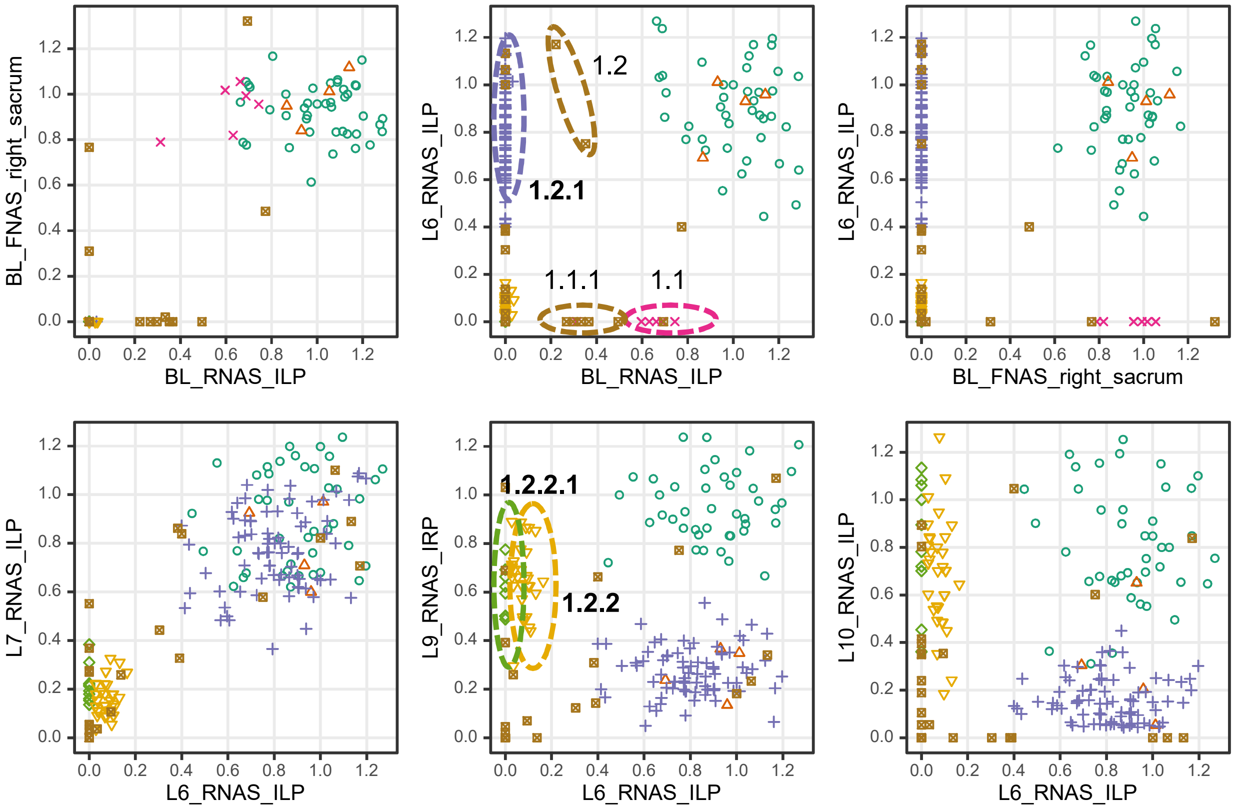

Supplementary Figure 18 (continued)

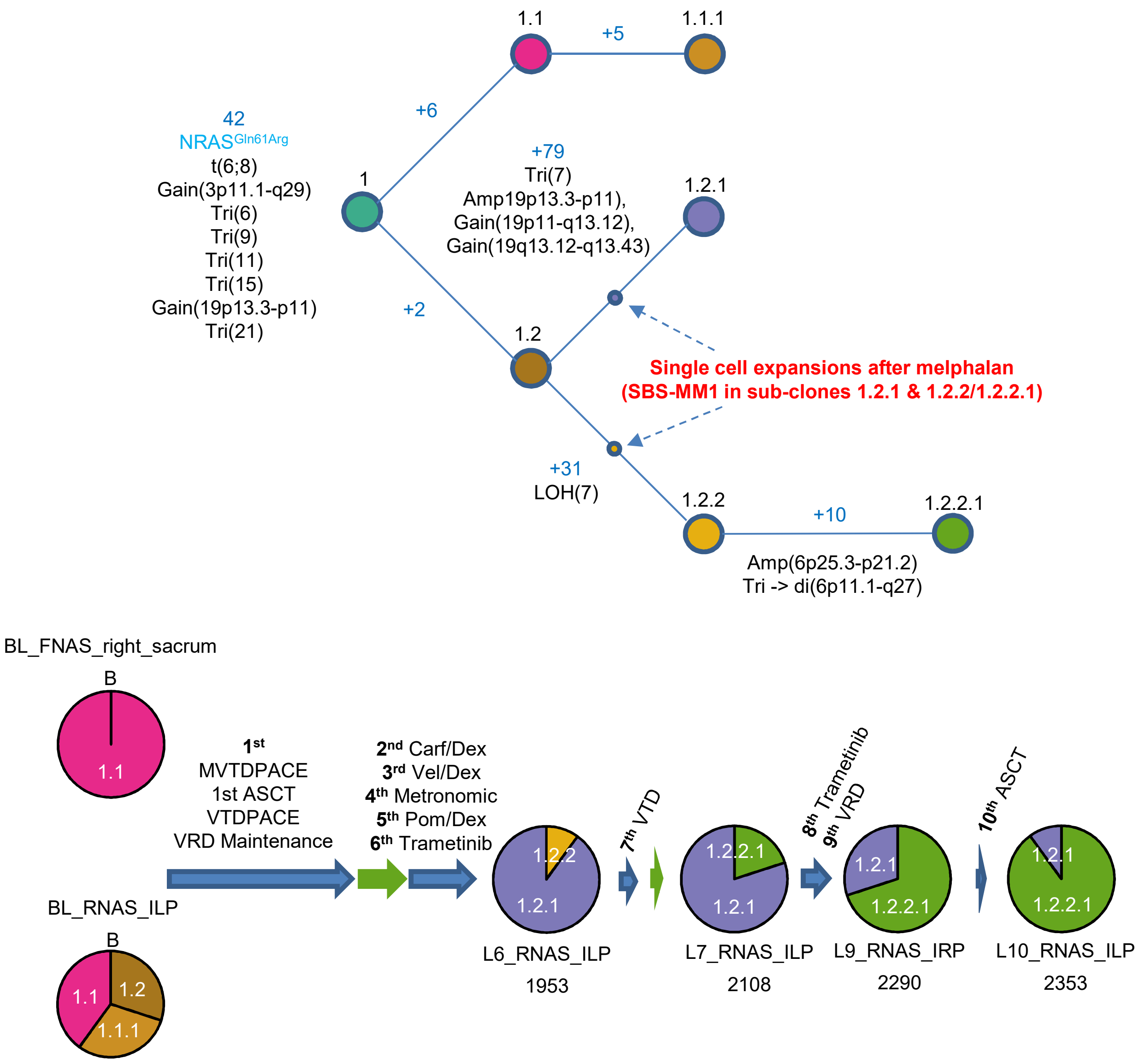

Supplementary Figure 19: Cancer clonal fraction plots, oncogenetic tree, and pie charts for the sub-clonal composition alongside treatment information for patient #17. A detailed legend is provided in Suppl. Fig. 2. Source data are provided as a Source Data file.

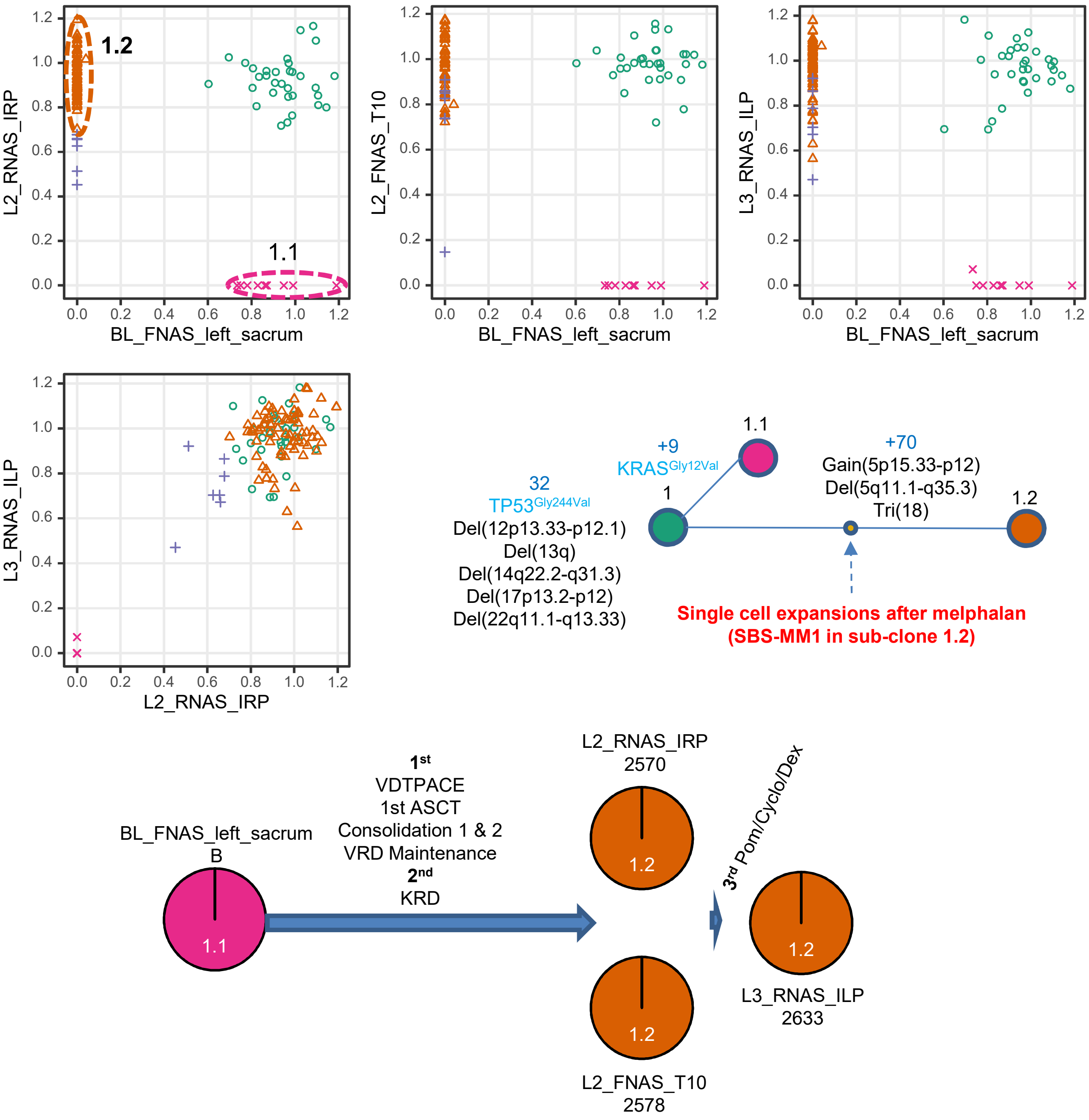

Supplementary Figure 20: Cancer clonal fraction plots, oncogenetic tree, and pie charts for the sub-clonal composition alongside treatment information for patient #18. A detailed legend is provided in Suppl. Fig. 2. Source data are provided as a Source Data file.

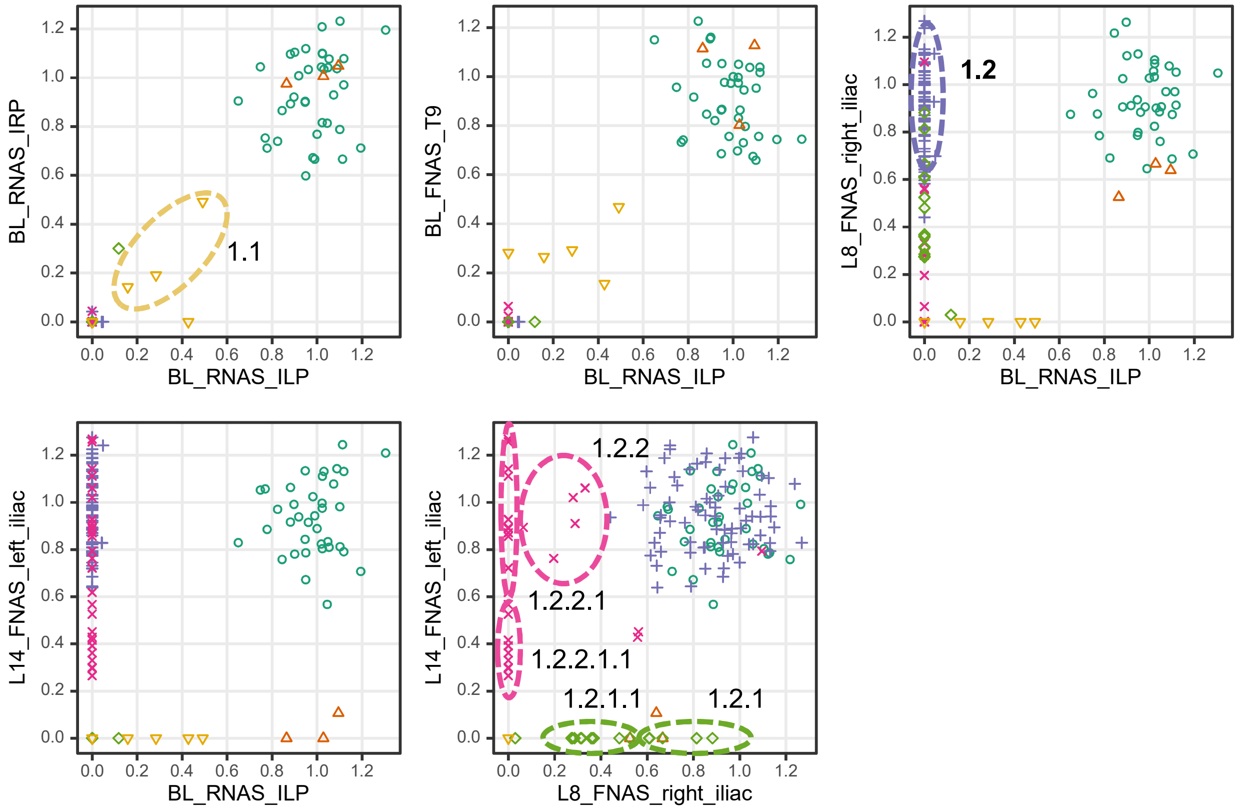

Supplementary Figure 20 (continued)

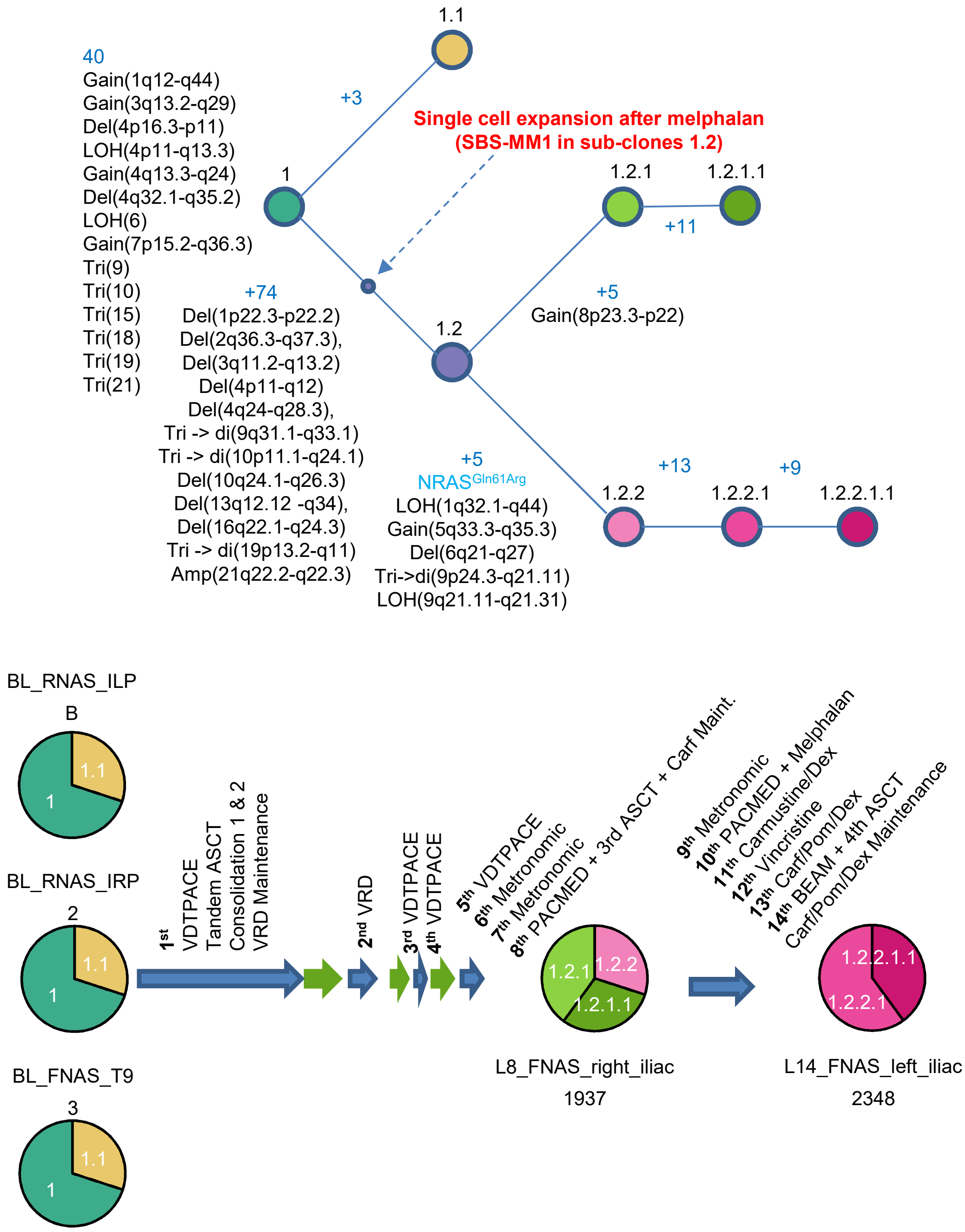

Supplementary Figure 21: Cancer clonal fraction plots, oncogenetic tree, and pie charts for the sub-clonal composition alongside treatment information for patient #19. A detailed legend is provided in Suppl. Fig. 2. Source data are provided as a Source Data file.

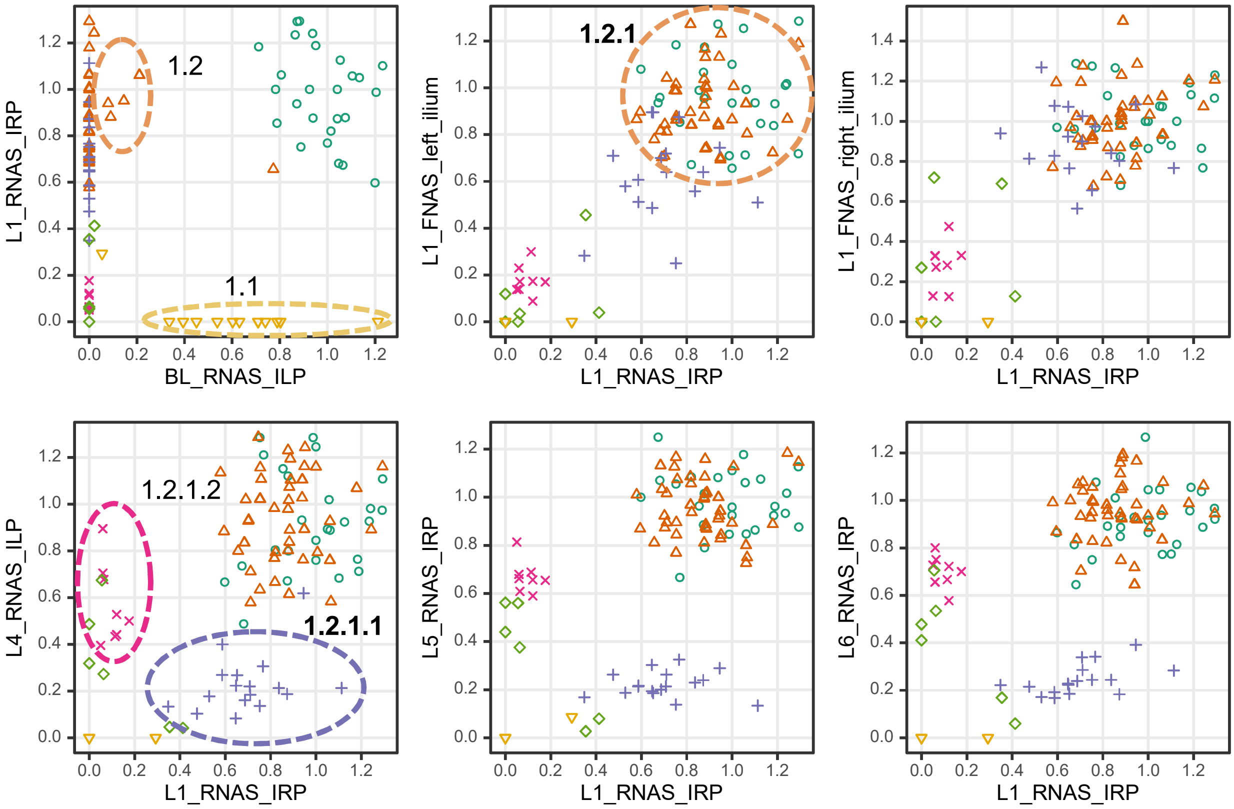

Supplementary Figure 21 (continued)

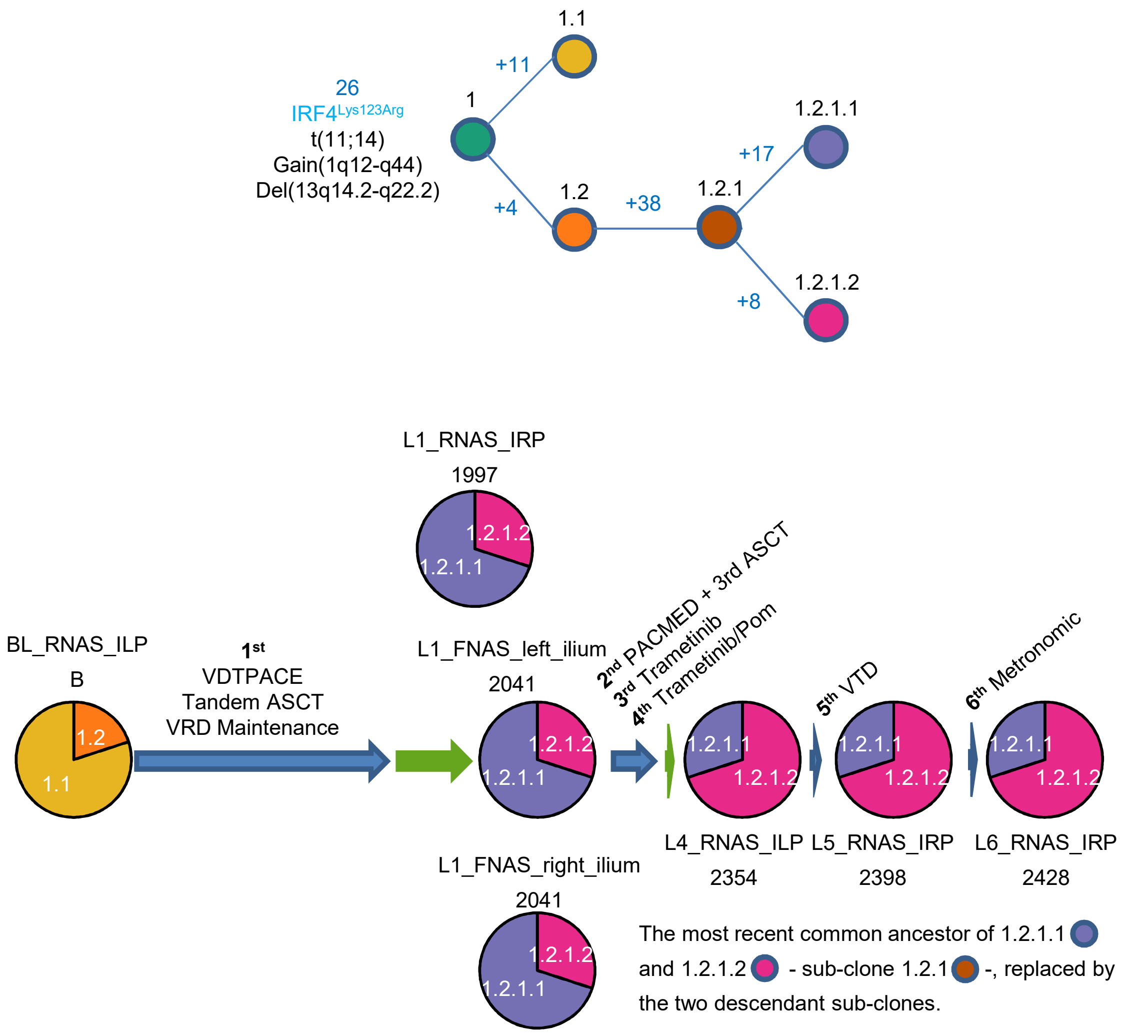

Supplementary Figure 22: Cancer clonal fraction plots, oncogenetic tree, and pie charts for the sub-clonal composition alongside treatment information for patient #20. A detailed legend is provided in Suppl. Fig. 2. Source data are provided as a Source Data file.

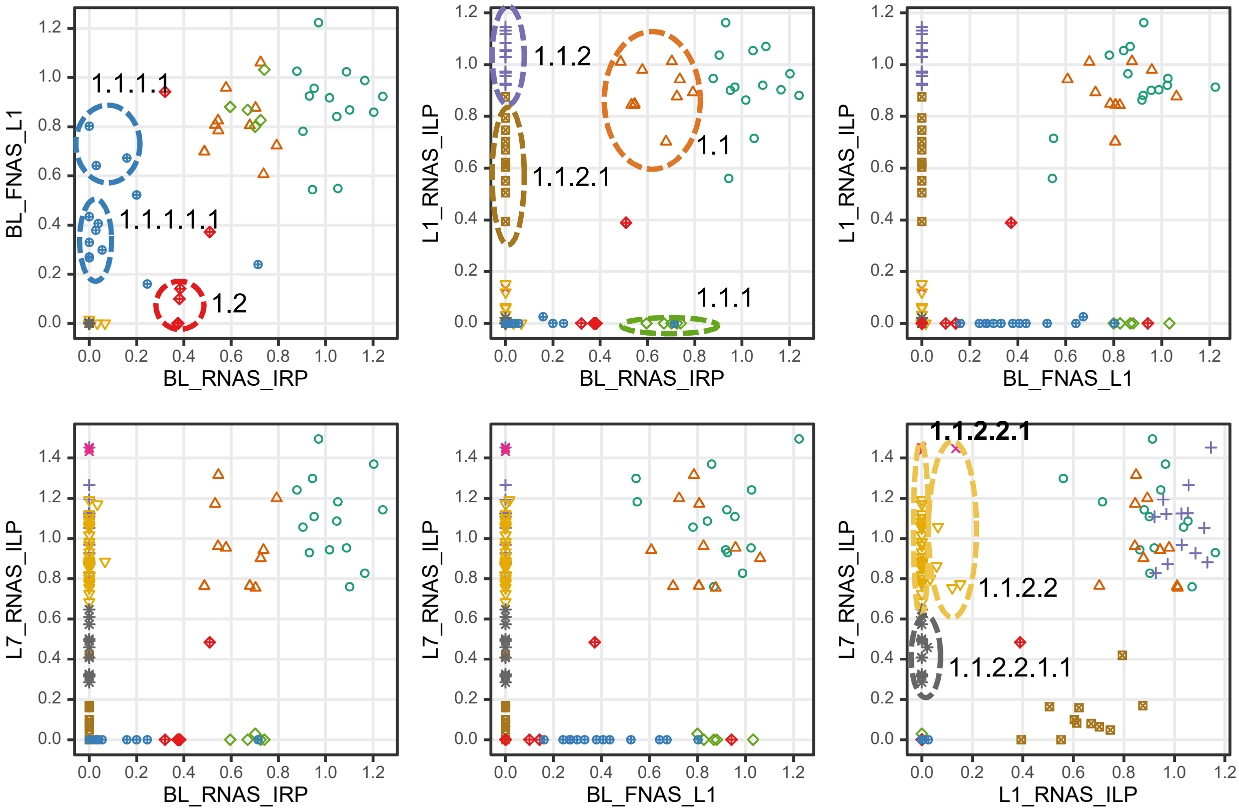

Supplementary Figure 22 (continued)

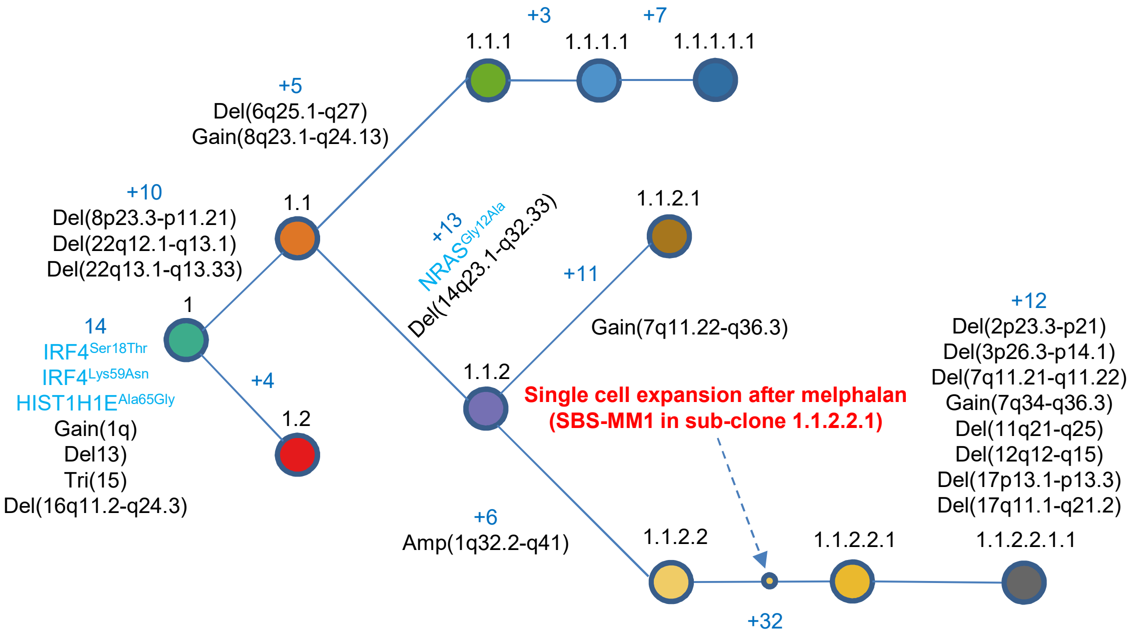

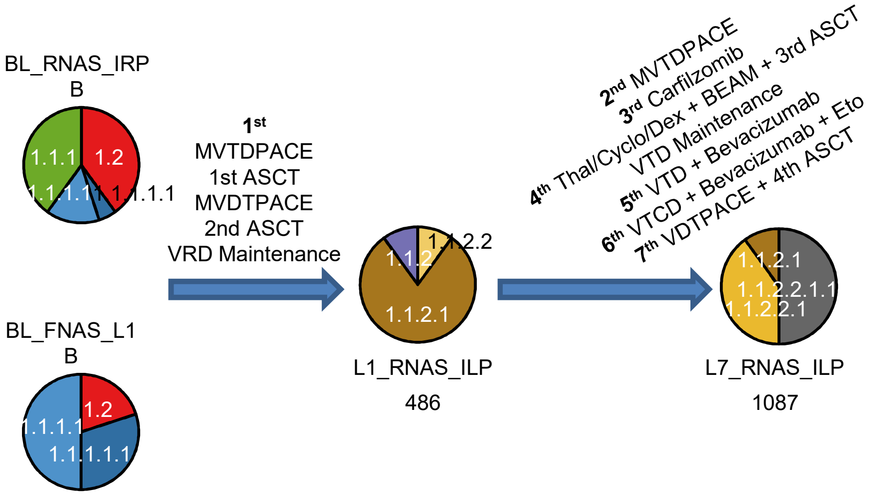

Supplementary Figure 23: Cancer clonal fraction plots, oncogenetic tree, and pie charts for the sub-clonal composition alongside treatment information for patient #21. A detailed legend is provided in Suppl. Fig. 2. Source data are provided as a Source Data file.

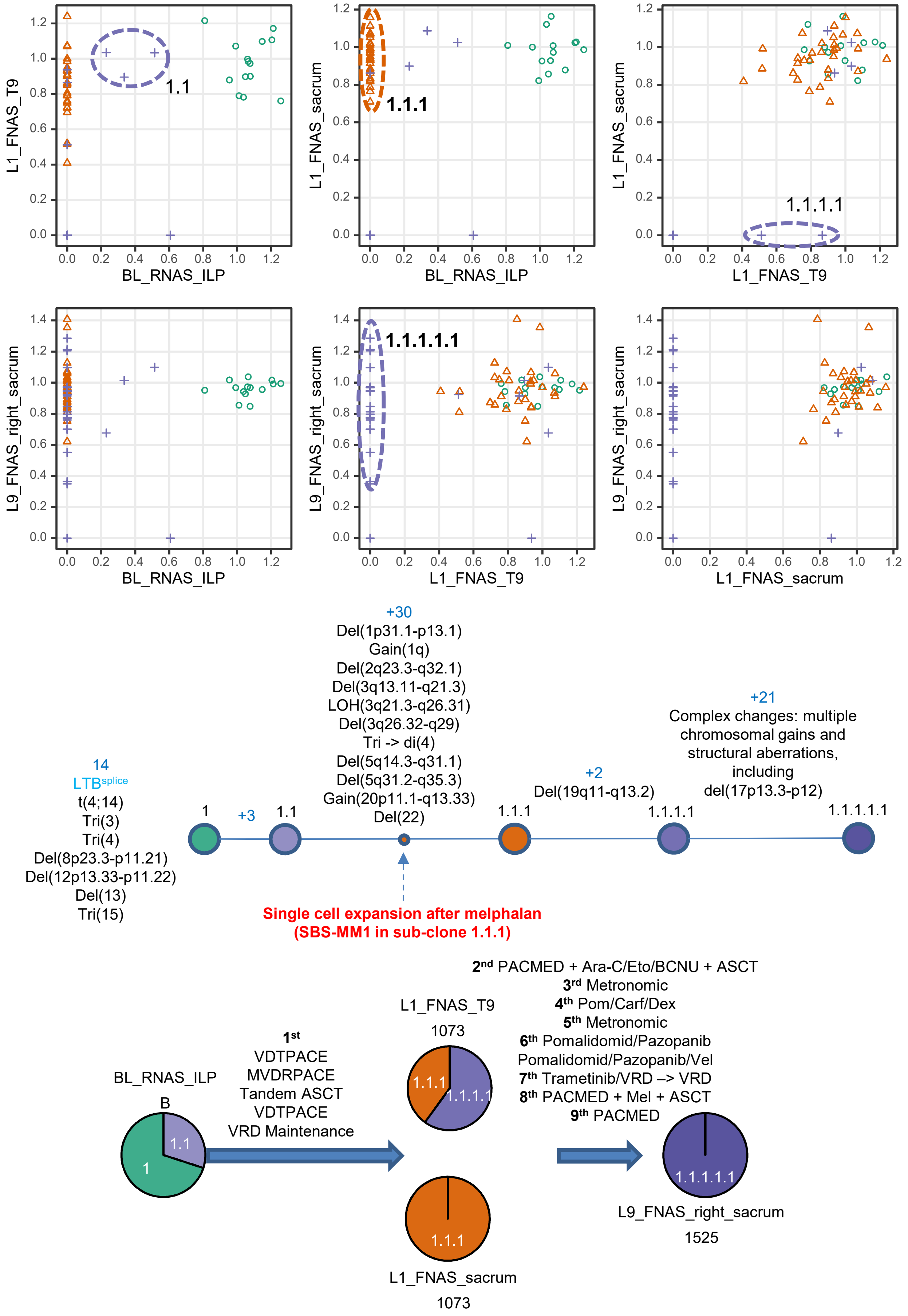

Supplementary Figure 24: Cancer clonal fraction plots, oncogenetic tree, and pie charts for the sub-clonal composition alongside treatment information for patient #22. A detailed legend is provided in Suppl. Fig. 2. Source data are provided as a Source Data file.

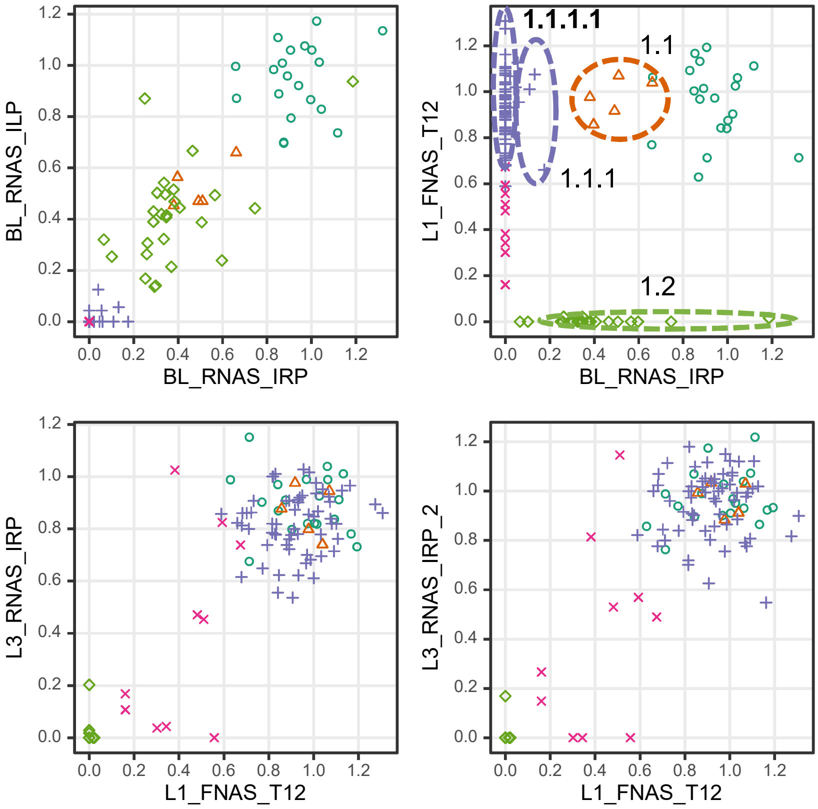

Sub-clone 1.1.1.1.1 appears as a minor sub-clone in L1_RNAS_IRP and further expands

Supplementary Figure 24 (continued)

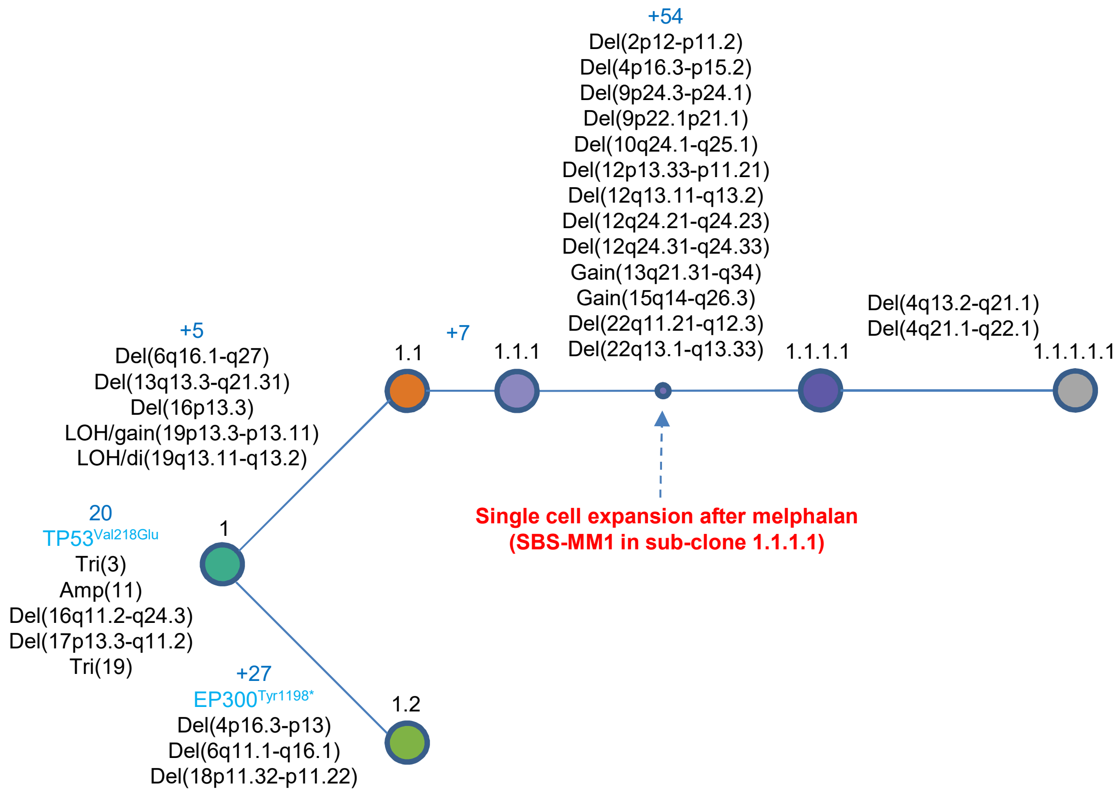

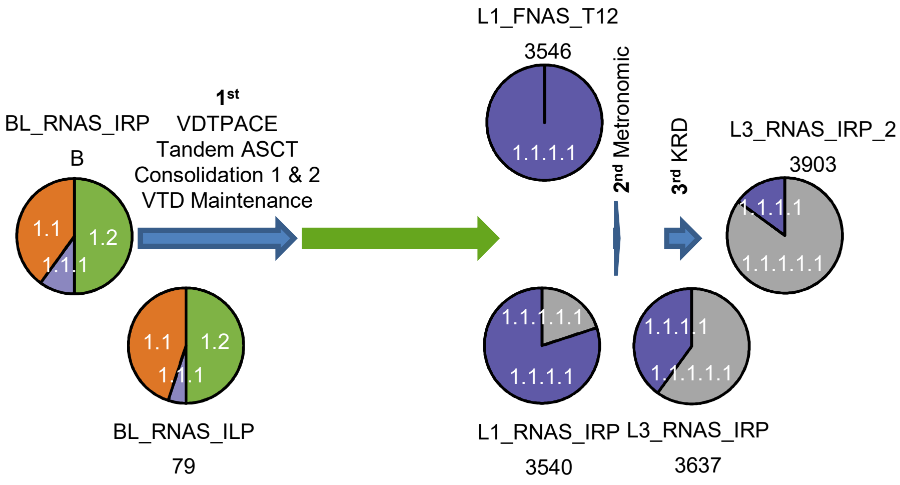

Supplementary Figure 25: Cancer clonal fraction plots, oncogenetic tree, and pie charts for the sub-clonal composition alongside treatment information for patient #23. A detailed legend is provided in Suppl. Fig. 2. Source data are provided as a Source Data file.

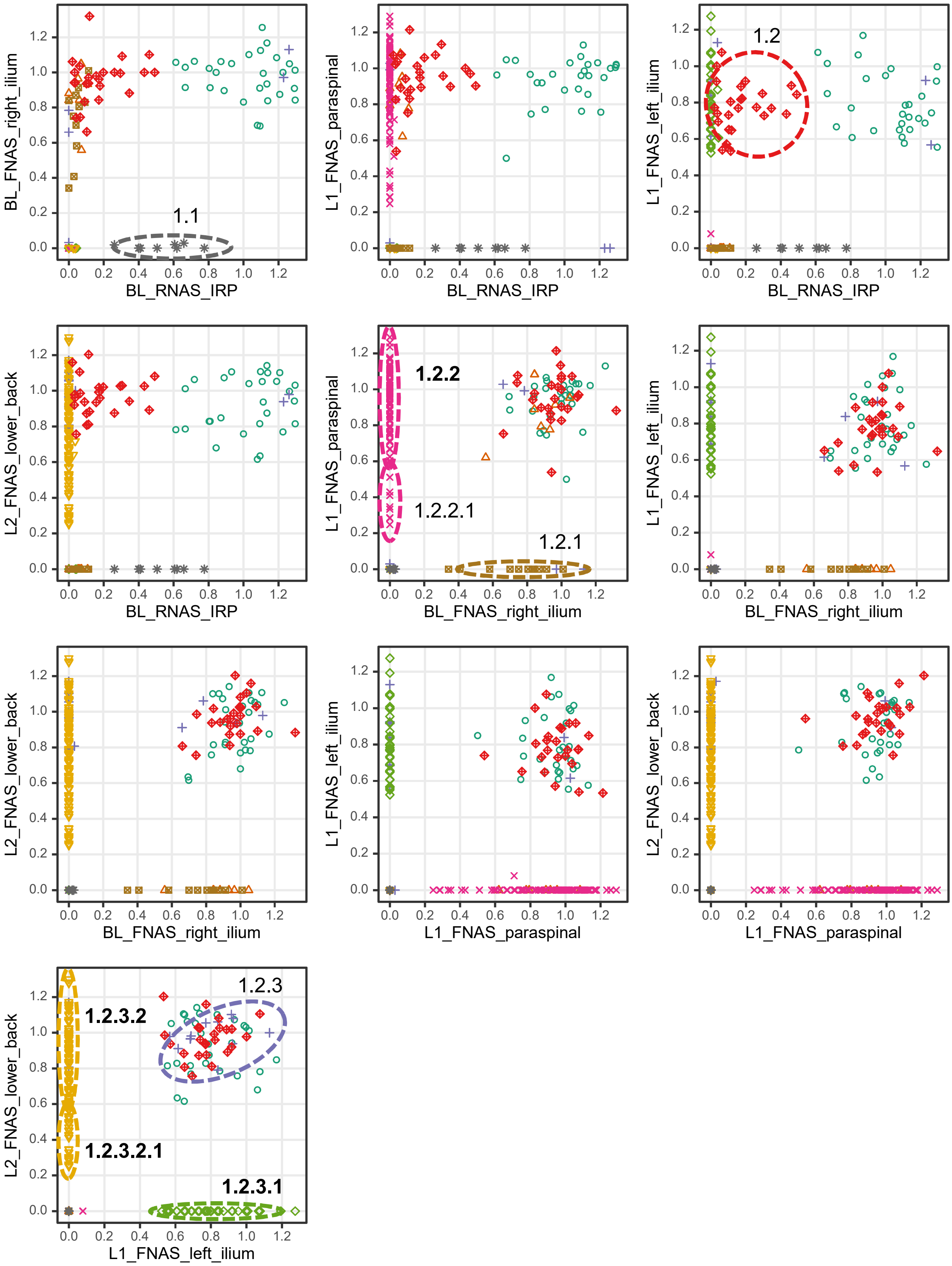

Supplementary Figure 25 (continued)

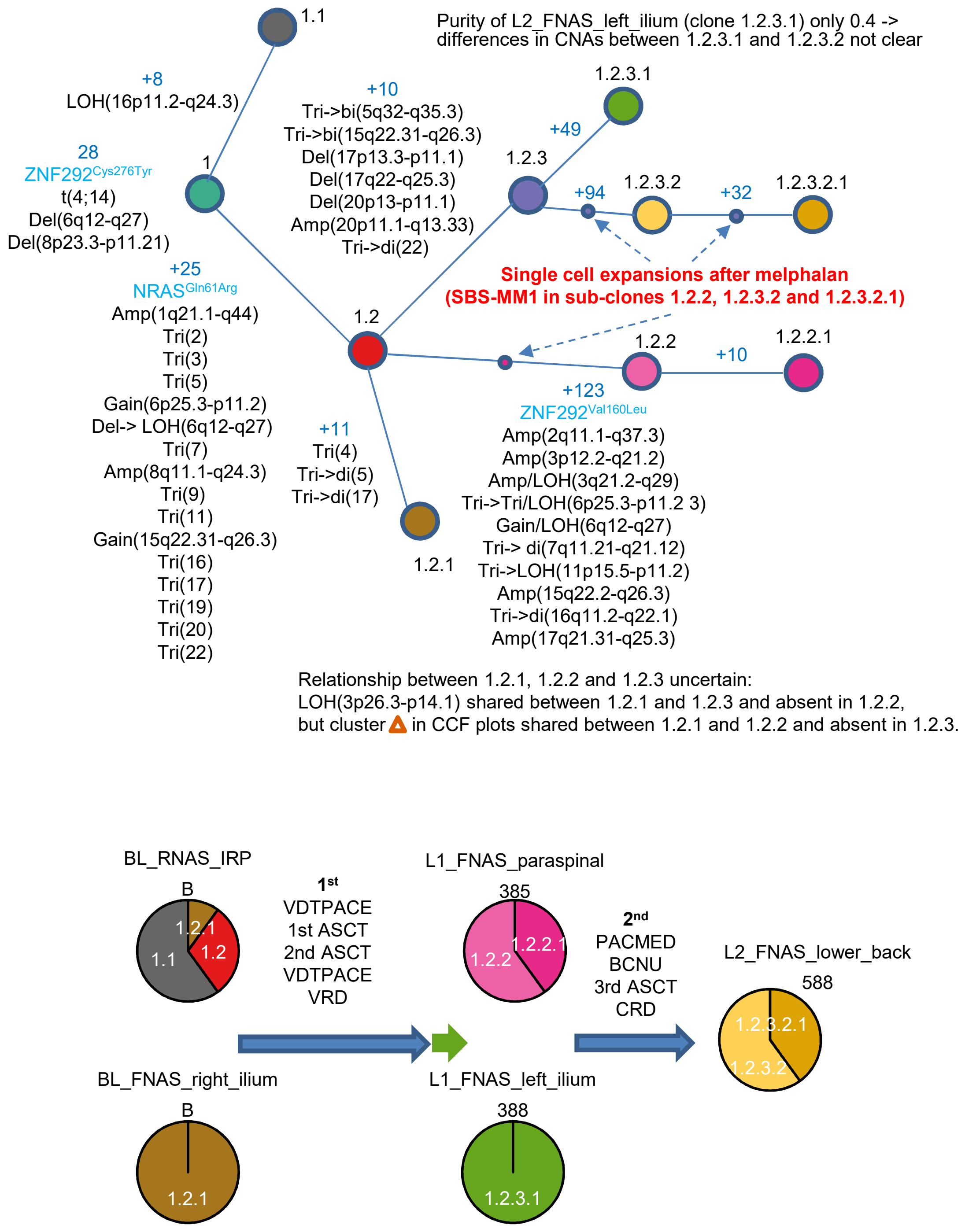

Supplementary Figure 26: Cancer clonal fraction plots, oncogenetic tree, and pie charts for the sub-clonal composition alongside treatment information for patient #24. A detailed legend is provided in Suppl. Fig. 2. Source data are provided as a Source Data file.

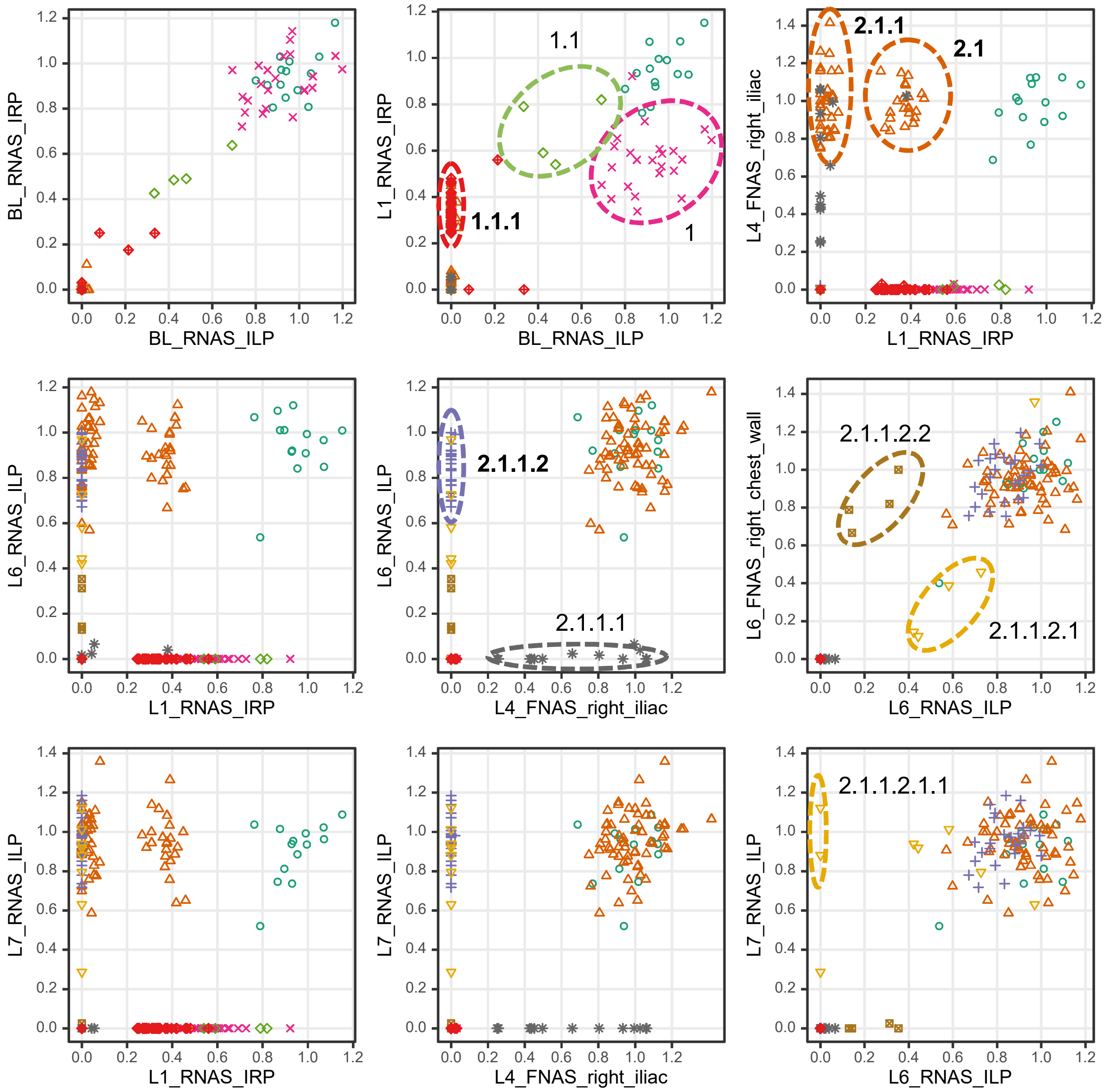

Supplementary Figure 26 (continued)

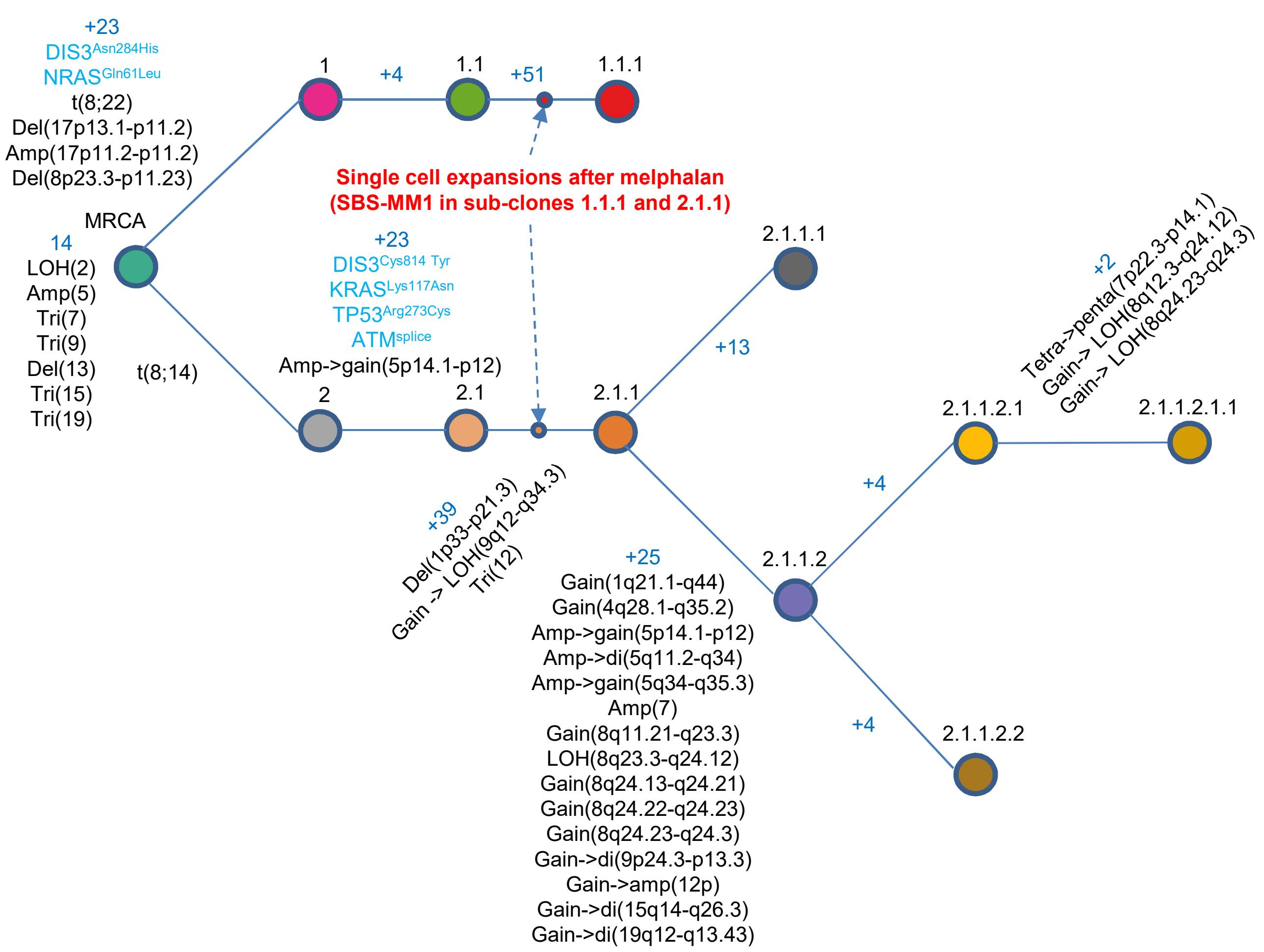

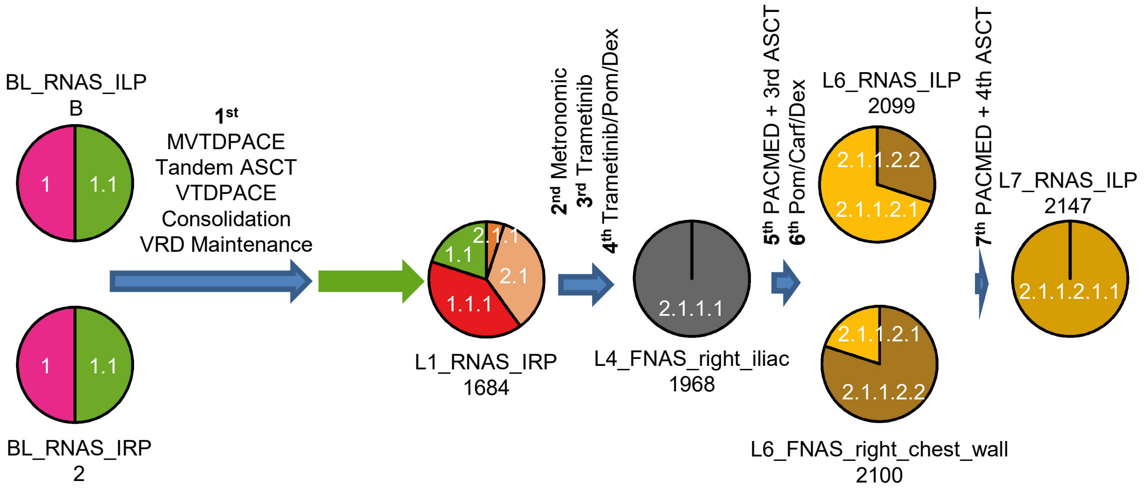

Supplementary Fig. 27: Alternating spatial clonal dominance of TP53-mutated sub-clones. PET-CT images for patient #13 at baseline and at two follow-up time points as well as the corresponding mock phylogenetic tree. In this patient, unique sub-clones were seen at distinct locations both at baseline and after relapse. The patient presented with three unique T*P53* double-hit events which are marked in red in the tree. Other known myeloma drivers are marked in bold. The length of branches corresponds to the time point of first appearance of respective clones. A timeline of treatment and sampling alongside a detailed mock oncogenetic tree is shown in Suppl. Fig. 15. Source data are provided as a Source Data file.

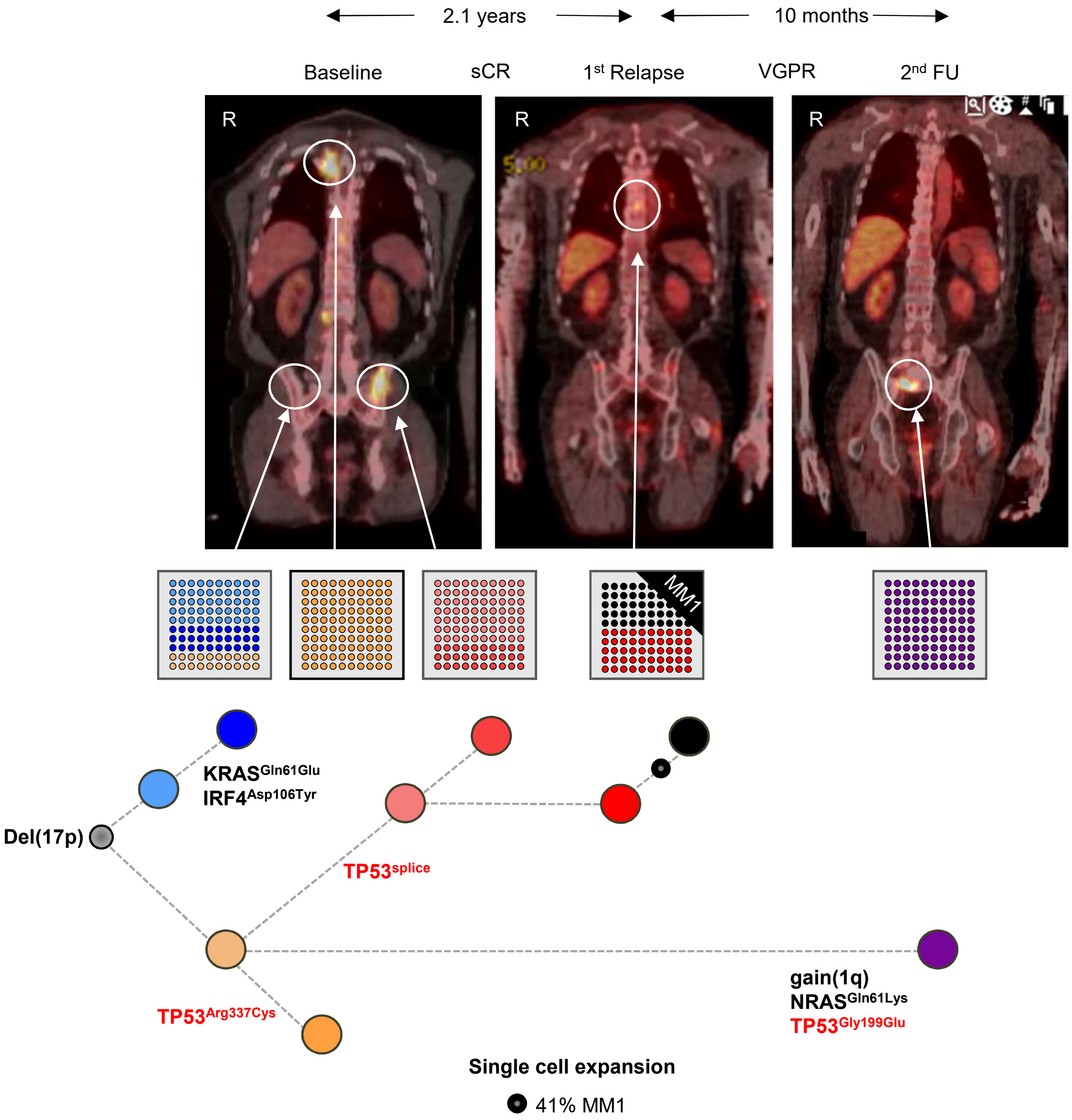

Supplementary Fig. 28: Concomitant selection of pre-existing disease and single-cell expansion. In patient #9 we observed selection of a pre-existing clone with a *KRAS* mutation at the right iliac crest and a concomitant expansion of a single-cell with an *NRAS* mutation at the right sacrum. The single-cell expansion was predicted based on the presence of the MM1 melphalan signature. Grey boxes/cards indicate that tumor data is not available for the respective timepoints and sites. The corresponding mock phylogenetic tree is shown on the right. The length of branches does not indicate the extent of differences between sub-clones. Dashed lines illustrate the origin/relationship of sub-clones/branches. A more detailed description of treatment and sub-clones is shown in Supplementary Fig. 11. Source data are provided as a Source Data file.

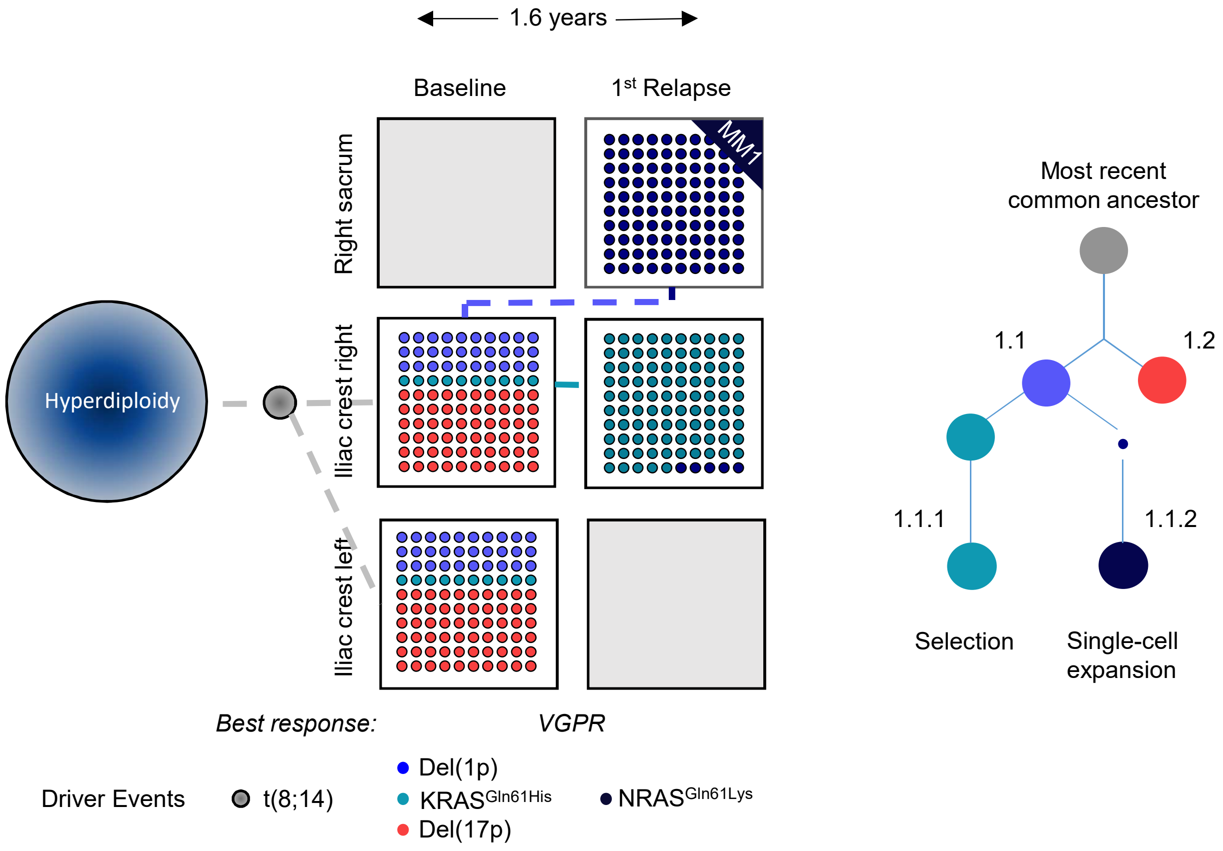

Supplement: Supplementary file 1 — Supplementary Information [file 41467_2022_32145_MOESM1_ESM.pdf]
