# Supplementary material for: The spatio-temporal evolution of multiple myeloma from baseline to relapse-refractory states
Source: Nat Commun. 2022 Aug 3;13:4517. doi: 10.1038/s41467-022-32145-y (PMC9349320; doi:10.1038/s41467-022-32145-y)
Supplement: Supplementary file 3 — Description to Additional Supplementary Information [file 41467_2022_32145_MOESM3_ESM.pdf]

Description of Additional Supplementary Files

Supplementary Data 1: Treatment and samples
