# Supplementary material for: The spatio-temporal evolution of multiple myeloma from baseline to relapse-refractory states
Source: Nat Commun. 2022 Aug 3;13:4517. doi: 10.1038/s41467-022-32145-y (PMC9349320; doi:10.1038/s41467-022-32145-y)
Supplement: Supplementary file 5 — Reporting Summary [file 41467_2022_32145_MOESM5_ESM.pdf]

nature portfolio

Corresponding author(s): Niels Weinhold

Last updated by author(s): Jul 18, 2022

Reporting Summary

Nature Portfolio wishes to improve the reproducibility of the work that we publish. This form provides structure for consistency and transparency in reporting. For further information on Nature Portfolio policies, see our Editorial Policies and the Editorial Policy Checklist.

Statistics

For all statistical analyses, confirm that the following items are present in the figure legend, table legend, main text, or Methods section.

n/a	Confirmed	
☐	☒	The exact sample size (*n*) for each experimental group/condition, given as a discrete number and unit of measurement
☐	☒	A statement on whether measurements were taken from distinct samples or whether the same sample was measured repeatedly
☐	☒	The statistical test(s) used AND whether they are one- or two-sided<br>*Only common tests should be described solely by name; describe more complex techniques in the Methods section.*
☐	☒	A description of all covariates tested
☐	☒	A description of any assumptions or corrections, such as tests of normality and adjustment for multiple comparisons
☐	☒	A full description of the statistical parameters including central tendency (e.g. means) or other basic estimates (e.g. regression coefficient) AND variation (e.g. standard deviation) or associated estimates of uncertainty (e.g. confidence intervals)
☐	☒	For null hypothesis testing, the test statistic (e.g. *F*, *t*, *r*) with confidence intervals, effect sizes, degrees of freedom and *P* value noted<br>*Give P values as exact values whenever suitable.*
☒	☐	For Bayesian analysis, information on the choice of priors and Markov chain Monte Carlo settings
☒	☐	For hierarchical and complex designs, identification of the appropriate level for tests and full reporting of outcomes
☒	☐	Estimates of effect sizes (e.g. Cohen's *d*, Pearson's *r*), indicating how they were calculated

Our web collection on statistics for biologists contains articles on many of the points above.

Software and code

Policy information about availability of computer code

Data collection	De-multiplexing of raw paired-end whole exome sequencing data was performed using bcl2fastq v2.20.0. Reads were aligned to the human genome reference GRCh37 release 75 (https://grch37.ensembl.org/index.html) using BWA-mem version 0.7.12. Sambamba v0.5.6 was used to sort and index bam files and to mark duplicates. Somatic single-nucleotide variants (SNVs) were called using MuTect v1.1.7 and Strelka v2.9.10 with default parameters. The intersection of SNVs identified by both variant callers was filtered using the fpfilter.pl script (https://github.com/ckandoth/variant-filter) with default parameters. After exclusion of variants located in immunoglobulin loci, we determined read counts for all mutations and samples per patient using the Rsamtools R package v1.24.0 and the following inclusion criteria: unique reads, coverage exceeding 20× in all samples of the patient, a mapping quality of at least 20 and base quality of at least 20 at the site of the variant. To maintain a conservative approach and avoid an overestimation of heterogeneity, we only included SNVs with a cancer clonal fraction (CCF) of ≥0.20 in at least one sample (corresponding to a clonal proportion of 20%) and called this SNP in the paired sample(s) if at least two variant reads were detected. For heterogeneous mutations, we performed manual somatic variant refinement using IGV v2.8.6 according to a published standard operating procedure. This filtered set of SNVs was annotated using SNPeff v4.3. Missense, nonsense, splice-site, and frameshift SNVs were defined as non-silent. For the analysis of spatio-temporal changes affecting known myeloma drivers, we only considered variants with a Combined Annotation Dependent Depletion (CADD) score >20. Ig and MYC translocations were identified using Manta v1.5.0 with default settings and the exome flag specified. All translocation calls were manually inspected in IGV. For annotation of translocations we used ANNOVAR v2017.07.01. Copy number aberrations were called using Sequenza v.2.1.2. For each sample, the accuracy of copy number calls was verified by manual inspection of LogR and BAF values for each aberration. To avoid overcalling heterogeneity we used a threshold of 5 Mb for global copy number aberration analyses. For the detection of deletions affecting MM driver genes we used a threshold of 1 Mb.
Data analysis	Description of heterogeneity: For the description of heterogeneity, we used the following terminology: we called mutations shared, if they were present in both samples with the same or similar cancer clonal fraction (CCF). We classified mutations with at least a three-fold difference in CCF as shared-differential ("shared-diff"). We called mutations unshared, if they were detectable in only one of the paired samples and discriminated between minor (CCF<60%) and major (CCF≥60%) mutations.<br>Signature analyses: We fitted mutations with the mutational signature single-base substitution (SBS)-MM1 (melphalan exposure) and the latest COSMIC reference (https://cancer.sanger.ac.uk/cosmic/signatures/SBS/) for SBS1, SBS2, SBS5, SBS8, SBS9, SBS13, and SBS18 using the

algorithmen mmsig v02.02.2020 (https://github.com/evenrus/mmsig) according to the authors' recommendations. Presence of SBS-MM1 was further confirmed using the mSigAct v0.9 signature presence test (https://genome.cshlp.org/content/suppl/2018/04/09/gr.230219.117.DC1) and a p value cut-off of 0.05.
Sub-clonal reconstruction: Clonal substructures were inferred using SciClone 1.1.0 with the filtered set of SNVs and default parameters, except for minimumDepth which was set to 50. For the manual design of mock phylogenetic trees, the output of SciClone was further interpreted after inclusion of copy number data. Sub-clones were defined based on SciClone clusters and presence of at least two mutations or at least one copy number aberration.
Statistical analyses: Analyses were carried out using the R software package 3.6.0. Group comparisons of continuous variables were done using the Mann-Whitney Wilcoxon test for independent groups. Differences in treatment responses and evolution patterns between groups was assessed using Fisher's exact test.

For manuscripts utilizing custom algorithms or software that are central to the research but not yet described in published literature, software must be made available to editors and reviewers. We strongly encourage code deposition in a community repository (e.g. GitHub). See the Nature Portfolio guidelines for submitting code & software for further information.

Data

Policy information about availability of data

All manuscripts must include a data availability statement. This statement should provide the following information, where applicable:

- Accession codes, unique identifiers, or web links for publicly available datasets
- A description of any restrictions on data availability
- For clinical datasets or third party data, please ensure that the statement adheres to our policy

The raw WES dataset has been deposited in the dbGAP database under accession number phs2625.v1 [https://www.ncbi.nlm.nih.gov/projects/gap/cgi-bin/study.cgi?study_id=phs002625.v1.p1]. Due to individual privacy concerns, the data are available under restricted access. Access may be requested by permanent employees of their institution at a level equivalent to a tenure-track professor or senior scientist with responsibilities such as laboratory administration and oversight. The requests are managed by the Data Access Committee of the NCI and after approval access is permitted for 12 months. The remaining data are available within the Article, Supplementary Information or Source Data file.

Field-specific reporting

Please select the one below that is the best fit for your research. If you are not sure, read the appropriate sections before making your selection.

☒ Life sciences ☐ Behavioural & social sciences ☐ Ecological, evolutionary & environmental sciences

For a reference copy of the document with all sections, see nature.com/documents/nr-reporting-summary-flat.pdf

Life sciences study design

All studies must disclose on these points even when the disclosure is negative.

Sample size	All samples with sufficient material were included.
Data exclusions	In order to validate our recent findings, patients which were included in our previous analysis of spatial heterogeneity (Rasche et al., Nat. Commun 2017) were not considered for comparisons of paired samples. Therefore, we excluded paired samples from patients #10, #13, #16, and #23 (baseline) as well as #9, #12, #22 and #24 (treated).
Replication	Due to limited material, whole-exome sequencing of this unique spatio-temporal sample set was performed once and not repeated. We replicated our recent finding that focal lesions have a distinct genetic makeup. The raw sequencing data are deposited in the publicly available dbGAP database and the remaining analysed data are available within the Article, Supplementary Information or Source Data file, in order to make it possible to reproduce our findings.
Randomization	In this restrospective study, we compared genomic data from samples, which had been collected before and during/after treatment by the treating physicians within the clinical routine. Furthermore, our molecular study was not a clinical trial. Hence, no randomization was performed.
Blinding	The analysis of changes in the sub-clonal architecture during treatment and the comparison of paired samples were performed independent of and blinded to clinical baseline parameters and endpoints.

Reporting for specific materials, systems and methods

We require information from authors about some types of materials, experimental systems and methods used in many studies. Here, indicate whether each material, system or method listed is relevant to your study. If you are not sure if a list item applies to your research, read the appropriate section before selecting a response.

Materials & experimental systems

n/a	Involved in the study
☒	☐ Antibodies
☒	☐ Eukaryotic cell lines
☒	☐ Palaeontology and archaeology
☒	☐ Animals and other organisms
☐	☒ Human research participants
☒	☐ Clinical data
☒	☐ Dual use research of concern

Methods

n/a	Involved in the study
☒	☐ ChIP-seq
☒	☐ Flow cytometry
☒	☐ MRI-based neuroimaging

Human research participants

Policy information about studies involving human research participants

Population characteristics	We included 24 multiple myeloma patients who had been treated at the University of Arkansas for Medical Sciences between 2003-2017. The patients were enrolled in total therapy protocols or treated with total therapy-like frontline regimen including multi-agent induction therapy, stem cell transplantation, and intensified maintenance therapy. Type of treatment and treatment lines are shown in Supplementary Table 1. Patients' characteristics, including gender, molecular subtype, and risk according to the GEP70, as well as the type of samples and the time points of sample collection are shown in Figure 1. Age at enrolment is shown in Supplementary Table 2. Nine patients were female, 15 patients were male. Median age at enrolment was 60 years and 5/24 patients had high risk according to the GEP70 risk classifier.
Recruitment	We included patients who had been treated with total therapy protocols or total therapy-like frontline regimen including multi-agent induction therapy, stem cell transplantation, and intensified maintenance therapy and for whom sufficient available material from baseline and relapse samples from the iliac crest as well as at least one focal lesion sample were available for whole exome sequencing.
Ethics oversight	The study was approved by the institutional review board of the University of Arkansas for Medical Sciences (#02815), and all patients signed written consent in accordance with the Declaration of Helsinki.

Note that full information on the approval of the study protocol must also be provided in the manuscript.
